# Dissecting the links between reward and loss, decision-making, and self-reported affect using a computational approach

**DOI:** 10.1371/journal.pcbi.1008555

**Published:** 2021-01-08

**Authors:** Vikki Neville, Peter Dayan, Iain D. Gilchrist, Elizabeth S. Paul, Michael Mendl

**Affiliations:** 1 Centre for Behavioural Biology, School of Veterinary Science, University of Bristol, Langford, United Kingdom; 2 Max Planck Institute for Biological Cybernetics, Tübingen, Germany; 3 School of Psychological Science, University of Bristol, Bristol, United Kingdom; Dartmouth College, UNITED STATES

## Abstract

Links between affective states and risk-taking are often characterised using summary statistics from serial decision-making tasks. However, our understanding of these links, and the utility of decision-making as a marker of affect, needs to accommodate the fact that ongoing (e.g., within-task) experience of rewarding and punishing decision outcomes may alter future decisions and affective states. To date, the interplay between affect, ongoing reward and punisher experience, and decision-making has received little detailed investigation. Here, we examined the relationships between reward and loss experience, affect, and decision-making in humans using a novel judgement bias task analysed with a novel computational model. We demonstrated the influence of within-task favourability on decision-making, with more risk-averse/‘pessimistic’ decisions following more positive previous outcomes and a greater current average earning rate. Additionally, individuals reporting more negative affect tended to exhibit greater risk-seeking decision-making, and, based on our model, estimated time more poorly. We also found that individuals reported more positive affective valence during periods of the task when prediction errors and offered decision outcomes were more positive. Our results thus provide new evidence that (short-term) within-task rewarding and punishing experiences determine both future decision-making and subjectively experienced affective states.

## Author summary

Affective states, such as happiness, are key to well-being. They are thought to reflect characteristics of the environment such as the availability of reward and the inevitability of punishment. However, there is a lack of agreement about: (i) the time scales over which these characteristics are measured; (ii) how and in what combinations actual or expected outcomes influence affect; (iii) how affect itself influences decision-making. A particular stance on the last issue underpins the judgement bias task, which, by measuring an individual’s willingness to make ‘optimistic’ or ‘pessimistic’ choices that are rendered risky by perceptual ambiguity, is one of the few cross-species tests for affect. Here we apply a novel computational analysis to a novel judgement bias task to examine all three issues. We reveal a rich interplay between affect and rewards, punishments, and uncertainty.

## Introduction

There is a traditional gulf in the field of decision-making between one-shot tasks, in which each trial is independent of the others (i.e., there is assumed to be no influence of previous outcomes or stimuli on current decisions), and ongoing tasks, in which individuals are supposed to apply what they learn from the consequences of their actions in earlier trials to later trials. Signal detection theory [1] is the most well-established framework for investigating the former, and reward- (or punishment-) based reinforcement learning [2] for the latter. In reality, the distinction is blurred—adaptation paradigms use repeated stimulus presentations to ‘set’ the state for one-shot psychophysical examinations [3]; participants perform exquisite Bayesian learning in stop signal reaction time tasks, despite the instructed independence of the trials [4].

This distinction is particularly pertinent in research where decisions on serial trials yielding reward or loss/punishment are used to probe the influence of affective states and disorders on risk-taking and reward sensitivity. The resulting decision-making profiles are proposed as markers of these states (e.g., Iowa Gambling task [5–7]; Balloon Analogue Risk Task [8–10]; Reward Responsiveness Task [11, 12]; Judgement Bias Task [13–15]). In most studies, decision-making data across test sessions are summarised into single statistics such as the number of cards selected from more favourable decks, the average number of ‘balloon’ pumps, the overall response bias as a marker of reward responsiveness, or the proportion of ‘optimistic’ responses. However, each trial within a task yields rewards or losses/punishments with the potential to influence the participants’ performance on future trials. Moreover, an individual’s ongoing experience of rewards and punishers (e.g., [16–20]) and their predictability (e.g., [21–23]) are considered to be fundamental determinants of affective states. Therefore, within-task experience may alter local affective states and influence future decisions and, ultimately, summary statistics of task performance, with knock-on effects for our understanding of the relationship between affect and decision-making and the utility of decision-making markers of affective states or disorders.

Here we use a judgement bias task as an example paradigm in which to investigate what characteristics of past within-task decisions and outcomes influence affective state, and how they influence present choices, either directly, or indirectly, via changes in affective state. Answering these questions will provide new fundamental information about how rewarding and punishing experiences influence affect and decision-making across short timescales, from seconds to a number of minutes. It will also further inform use of the judgement bias task as a method for assessing both human and animal affective states.

The judgement bias task was originally designed [13], and has since been widely used [14, 15], as a translational paradigm in which decision-making under ambiguity could be used as an indicator of an individual’s affective state. In the task, participants are trained to discriminate between two stimuli and make different responses to each (typically ‘go’ or ‘stay’/‘no-go’) in order to either obtain a reward or avoid a punisher. Participants are then presented with probe stimuli that are ambiguous by lying intermediate between the trained stimuli, and their responses are recorded. In typical go/no-go tasks, executing the response associated with the trained rewarded stimulus, which is deemed the ‘optimistic’ response in the judgement bias literature, is risky in that it could result in either a reward or punisher, while executing the response associated with the trained punished stimulus (deemed ‘pessimistic’) is ‘safe’ in that it guarantees avoidance of the punisher, but also removes the possibility of winning a reward.

Decision-making in the judgement bias task, particularly trials with perceptually ambiguous stimuli, is considered to be indicative of a longer timescale notion of affective state, or ‘mood’. Specifically, more ‘optimistic’ decision-making, as indicated by an excess proportion of ‘optimistic’ as opposed to ‘pessimistic’ decisions made across a test session, is typically associated with more positively valenced affect [14, 15, 24]. This decision-making profile is hypothesised to reflect approximate sufficient statistics about the characteristics of the individual’s interaction with and experience of the rewarding and punishing features of their environment [22, 25, 26]. These statistics could encompass such things as past provision of rewards and punishers or the extent to which these were predicted, and could affect how the individual makes decisions by, for instance, changing their prior expectations, or their sensitivities to reward and punishment [17, 22, 25–28]. To illustrate, consider an individual in a chronically reward-barren environment. Such an environment has been proposed to induce a depression-like state which is sometimes associated with a reduced reward valuation (i.e., anhedonia—reduced sensitivity to the value of rewards; [29, 30]) and a reduced expectation of future rewards, and hence should result in more ‘pessimistic’ decision-making in the judgement bias task [17, 25–28].

While these statistics might reflect long-term experience prior to the task, they might also reflect short-term and ongoing experience within the task. That is, the judgement bias task is ostensibly a series of independent one-shot psychophysical choices, each open to influence from longer-term affective state as described above. However, during performance of the task, participants accrue rewarding or punishing outcomes as a result of their decisions. These outcomes might in turn alter the very affective state that the task is designed to measure. Clearly, the influence of the consequences of previous decisions in the task on subsequent affect and choices needs to be investigated.

In the present study, we examined this relationship between psychophysical decision-making, decision-outcomes and self-reported affect in human participants using a novel ‘go’/‘stay’ judgement bias task design in which either reward or loss magnitude was systematically varied across trials, so that there were epochs of high and low value potential outcomes. We investigated the impact of on-going reward and loss experience on decisions on each trial, and on self-reported affective state at intervals during the task. Choice and/or latency are the typical outcome measures in judgement bias tasks; we further exploited these by constructing a computational model of the task that allowed us to model the course of decision-making in detail [31, 32] and thereby investigate the latent, and potentially more fundamental, variables underlying the relationship between reward and loss experience, affect, and decision-making in this task. This study hence provides a comprehensive investigation of the interplay between affect, outcomes, and decision-making by exploring the links between reward and loss experience and affective state, reward and loss experience and decision-making, and affective state and decision-making simultaneously.

The model considers the task as a partially observable Markov decision process (POMDP). In this, on each trial, participants transition through a two-dimensional state space, which bears some resemblance to a discretised version of a drift-diffusion model: one dimension represents accumulated information about the presented stimulus, as informed by their observations, and the other dimension represents the (discretised) time elapsed on the trial. The probability that a participant executes a ‘go’ action (associated with avoiding the punished stimulus), the alacrity with which they do so, and the probability of executing the ‘stay’ action (associated with obtaining the rewarded stimulus) will depend on their transitions through this state space and their subjective value of occupying each state. This is determined by two sets of parameters. One set characterises task performance, and includes a *slope*, which represents the ability to discriminate the stimuli, and *lapse* parameters, which represent the propensity to make errors at either trained or ambiguous stimuli. These lead to the psychometric function [33]. The second set of parameters quantify time estimation, decision stochasticity, and finally biases towards or away from ‘optimistic’ decision-making. Because biases in decision-making may be influenced by experience prior to the task, but also by experience of decision outcomes during the task itself, we used additional parameters that mapped the influence of within-task experience to this bias parameter. These included the *average earning rate* (reflecting what a participant has learnt about their earnings from previous trial outcomes), *prediction error* (the difference between the actual and expected outcome on recent trials) and *squared prediction error* (magnitude of unpredictability of outcomes on recent trials). All of these have been implicated in affect and decision-making and/or the relationship between the two in a variety of ways (prediction errors: [21, 22, 34, 35]; average earning rate: [36, 37]). Critically, the model precisely quantifies the various factors determining behaviour that can then answer our specific research questions.

Our main hypothesis was that a less favourable within-task experience would induce more negatively-valenced affect and consequently more risk-averse decision-making. Specifically, we hypothesised that more negative prediction errors and a lower average earning rate (a weighted average of past outcomes) would reflect epochs in which the test environment is both relatively and absolutely unfavourable, thereby promoting more negatively-valenced affect [22, 25, 38], as assessed by a self-reported affect measure [21, 39, 40]. In turn, we predicted that the same factors predicted to induce negatively-valenced affect (i.e., more negative prediction errors and a lower average earning rate) would be associated with riskier decision-making.

In contrast, the literature does not suggest such clear predictions for positive prediction errors. They might be associated with more positively-valenced affect [21, 22], but given that they are also indicative of greater uncertainty, they have also been associated with more negatively-valenced affect [23, 41, 42]. Hence, we remained agnostic as to the direction of the effect of positive prediction errors on affect and decision-making, and to whether the direction or magnitude (i.e., unpredictability) of prediction errors would provide a better account of within-task variation in affect and decision-making.

We further hypothesised that overall more positive affective valence would be associated with an overall greater ‘optimistic’ bias/greater risk-seeking, based on previous research [30, 43, 44]. Given that losses can be more salient than gains [45–47], we investigated the possibility that all effects of past outcomes on affect might be stronger in the fluctuating loss condition than in the fluctuating reward condition.

## 1 Results

**Fig 1:**
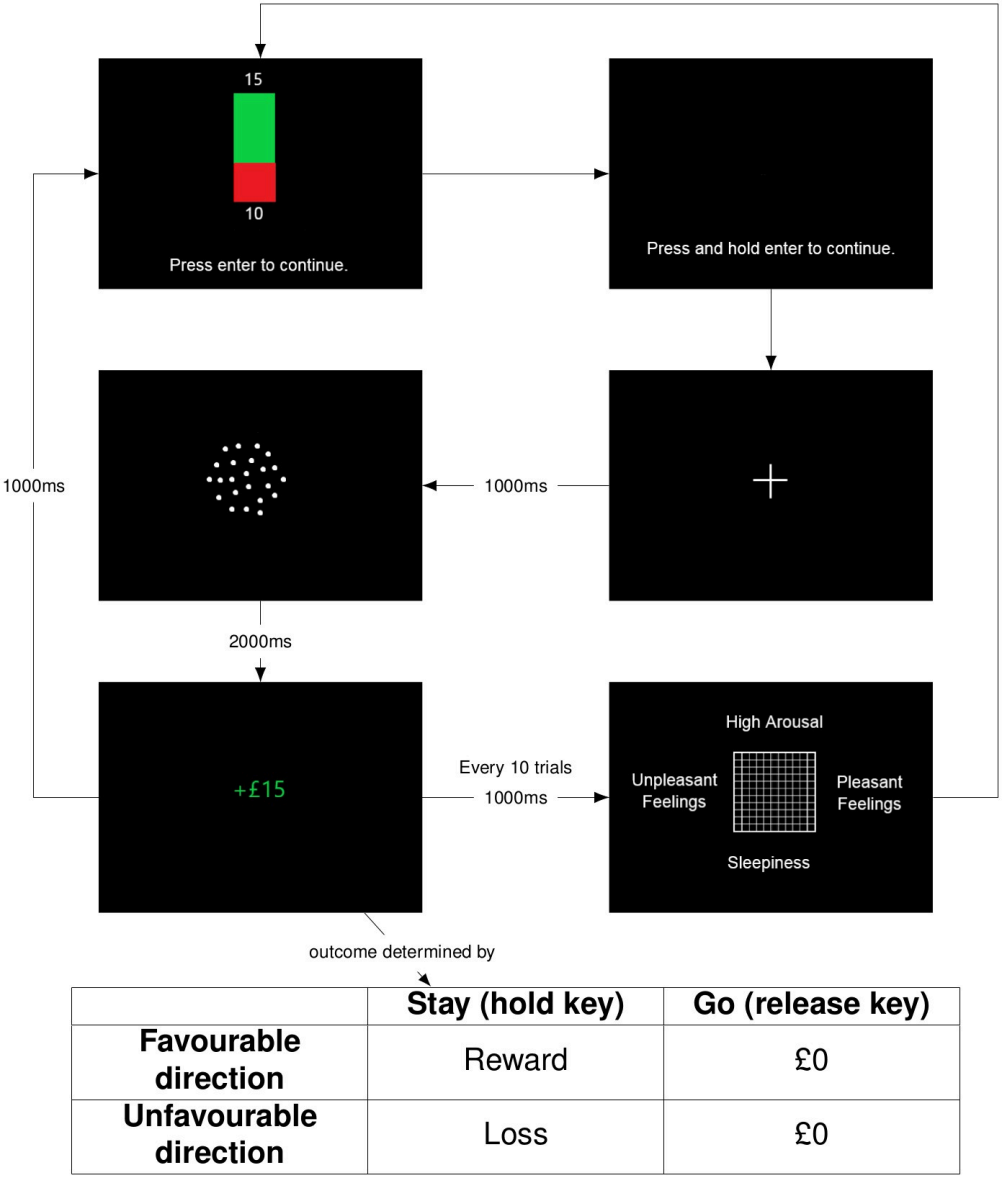
Task structure. Structure of the human monetary judgement bias test session: (1) participants are shown the potential outcomes of the ‘stay’ response and then must press ‘enter’; (2) participants are instructed to press and hold the ‘enter’ key; (3) participants are shown a fixation cross for 1000ms (3) participants are presented with a RDK for 2000ms during which they must either continue holding the ‘enter’ key (‘stay’) or release the ‘enter’ key (‘go’); (4) participants are shown the outcome of their action (which is also determined by the true direction of the RDK) for 1000ms (5) either the next trial starts or the participant is asked to complete an affect grid (after every 10 trials).

In order to examine the relationships between rewards and punishments, affective states and risky decision-making, we asked human participants to perform a novel version of a go/no-go judgement bias task (see Fig 1), whilst also providing reports on their affective states. The task involved participants choosing between a risky response (‘stay’; in which they continued to hold a key on a keypad for the 2s duration of the trial, resulting in either a reward or loss) or a safe response (‘go’; in which they released the key before the trial elapsed, resulting in neither a reward nor loss). Participants were informed about the reward or loss outcome of the risky response by the direction of motion of a random dot kinematogram (RDK) which varied in ambiguity. We analysed the choices/reaction times and affective reports using a new computational approach which is sensitive to subtle influences of past outcomes and predictions on present decisions and reports.

We start by laying out how our model captured trial-, subject-, and history-dependent choices in the task. This leads to a set of parameters that we then relate to the affect that the participants report during performance of the task.

The ultimate model involves a large number of parameters. We controlled the model complexity by adding only those that reduced the BIC score, which is a complexity-sensitive measure of the fit of the model to the subjects’ performance. We then compared the resulting BIC scores [48] along with those of another complexity measure, the AIC [49], across a final set of models (see S1 Appendix). To validate our procedure, we also checked that we could recover subjects’ parameters from data generated by the model, compared the results to those obtained using a quasi model-agnostic approach, and compared the model fit to an alternate model of judgement bias (see S1 Appendix).

### 1.1 Judgement bias

We first sought to identify the parameters which best accounted for variation in decision-making both across trials and between participants on the judgement bias task. We paid particular attention to participants’ (discretised) reaction times. Each model included various sets of parameters, the first of which characterised an individual’s decision-making in the absence of baseline or experience-dependent biases. These models are detailed in full in S1 Appendix. The raw experimental data are provided in S1 Data. Here, we describe the functional role of the parameters.

At the heart of the model is a simple psychometric function, which uses three participant-specific parameters: a *slope* (*σ*); and two *lapse rates* (λ_amb_ and λ_ref_), which turn the objective rewards and punishments into a subjective probability of choice. The use of two lapse rates is justified by model parsimony (ΔAIC = 316.650; ΔBIC = 192.125); we consider that it may arise from the application of a different strategy on cases when there could be very little doubt about the direction of motion. As uncertainty in time estimation is fundamental to the task, given that the trial will ‘timeout’ and the risky choice made, de facto, if the participants do not make a decision prior to the end of the trial, two parameters were included to encompass errors in time estimation; *ζ* and *ϕ*. These are respectively the shape and scale parameters of a gamma distribution representing the uncertainty about the interval (with mean *ζϕ* and variance *ζϕ*^2^). Finally, a parameter characterising decision stochasticity (*B*) was included to capture the extent to which decisions executed within a model state depended on the value of that state.

Central to the decision-making component of the judgement bias task is that ambiguous choices may be biased, in a participant-specific manner, in the risk-seeking/‘optimistic’ (‘stay’) or risk-avoidant/‘pessimistic’ (‘go’) directions. We characterised this tendency in general using just such a participant-specific, constant, *bias* parameter; $\beta_{0}^{\delta }$. We first assessed whether the parsimony of the model was improved by including this bias.

However, our current hypotheses concern the possibility that ‘optimistic’ or ‘pessimistic’ biases might not be fixed across all trials for a single participant, but rather that they might be modulated around a baseline by the ongoing experience that participants have *during* the task. We quantified experience in terms of a few key statistics: the *average reward rate* (${\bar{R}}_{n-1}$: reflecting what a participant has learnt about their earnings from previous trial outcomes), the *weighted prediction error* (*w*PE_*n*−1_: difference between actual and expected outcomes on recent trials), the *squared weighted prediction error* ($w{\text{PE}}_{n-1}^{2}$: magnitude of unpredictability of outcomes on recent trials), and the *outcome of the previous trial* *O*_*n*−1_. Then, we parameterised how these various experiential factors might modulate the baseline bias parameter, and assessed whether inclusion of these parameters would improve the model fit.

The best-fitting model included nine parameters: *σ*, λ_ref_, λ_amb_, *B*, $\beta_{0}^{\delta }$, $\beta_{\bar{R}}^{\delta }$, $\beta_{O}^{\delta }$, *ζ*, and *ϕ* (see S1 Appendix). This model was the AIC-best model, and the BIC-best model included all of these parameters except $\beta_{O}^{\delta }$ (see S1 Appendix). However, we conducted further analyses to assess whether this parameter should indeed be included in the model, which involved analysing whether the parameter estimate differed from zero using two separate approaches—these additional analyses justified inclusion of this parameter in the final model (see S1 Appendix).

**Fig 2:**
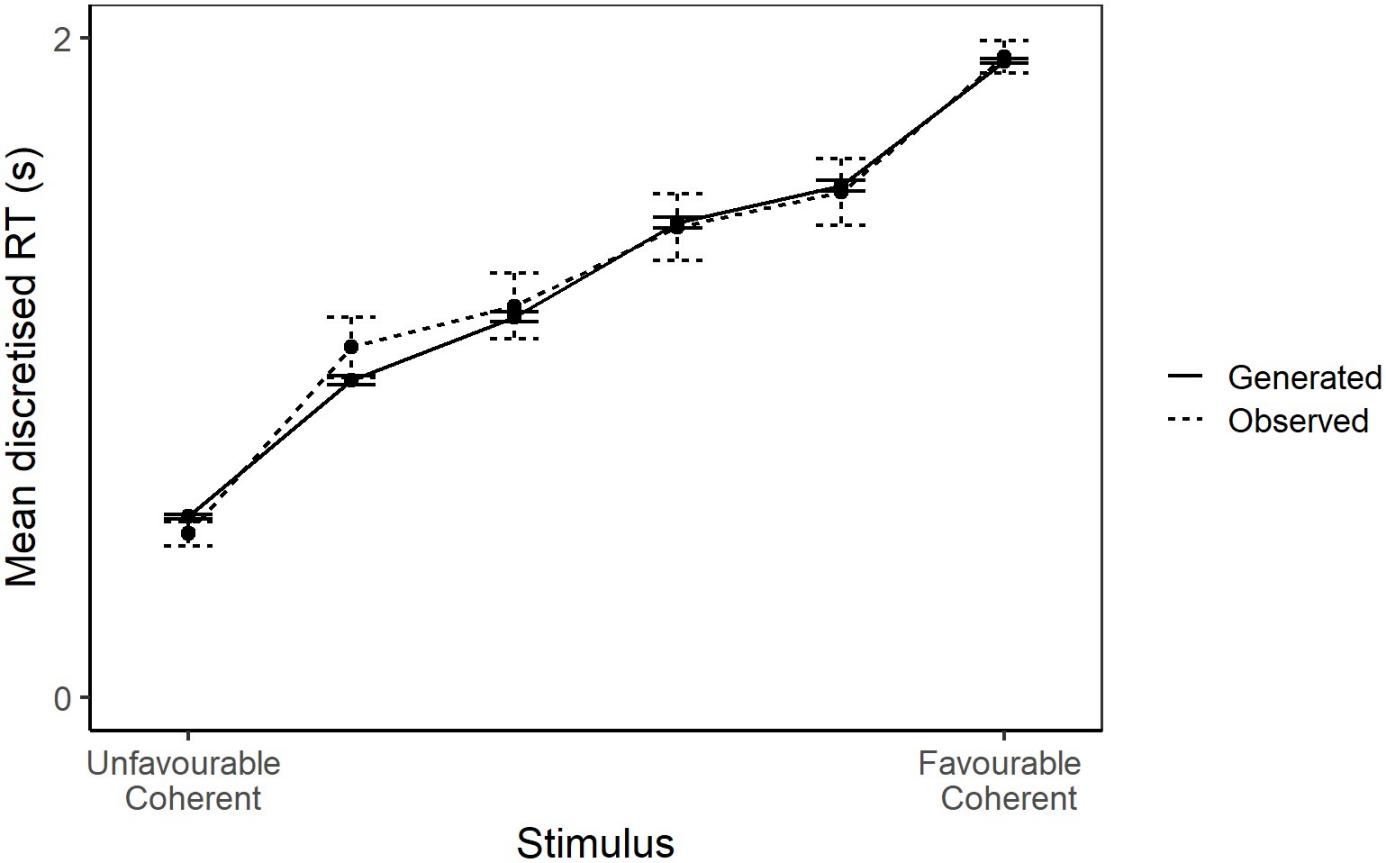
Observed vs. generated reaction time data. The mean discretised reaction time for each stimulus level for both the model-generated and observed judgement bias data. Error bars represent one standard error.

The model provides a good fit of discretised reaction time data (Fig 2). Furthermore, assessment of parameter recovery and comparison with alternate models supports the reliability of the parameter estimates (see S1 Appendix).

Following model selection, permutation tests were used to examine whether and how the parameter estimates differed from zero. As *ζ* and *ϕ* jointly determine the timeout probabilities, the mean timeout probability $\left(\frac{\Sigma_{0}^{T}P_{t;\text{timeout}}}{t}\right)$ was analysed instead of *ζ* and *ϕ* for a more intuitive interpretation of the results.

Estimates of the bias parameter were significantly greater than zero ($\beta_{0}^{\delta }$: mean±SE = 0.711 ± 0.141, p<0.001), while estimates of the influence of both $\bar{R_{n}}$ (the average earning rate) and *O*_*n*−1_ (the previous outcome) were significantly lower than zero ($\beta_{\bar{R}}^{\delta }$: mean±SE = −0.143 ± 0.042, p<0.001; $\beta_{O}^{\delta }$: mean±SE = −0.018 ± 0.006, p<0.001). Thus, participants were overall risk-seeking on the task, but were more risk-averse when they had experienced greater recent earnings and when the most recent outcome was more favourable.

### 1.2 Correlation between parameter estimates and reported affect

As a next stage of analysis, we considered whether there was a relationship between the model parameters that were fitted to the participants’ discretised reaction times and aspects of their reported affect (Table 1). More negative reported affective valence was significantly associated with a higher timeout probability ($\frac{\Sigma_{0}^{T}P_{t;\text{timeout}}}{t}$: LRT = 6.662, p = 0.010), and tended to be associated with a greater bias towards the ‘risky’ response ($\beta_{0}^{\delta }$: LRT = 3.526, p = 0.060). Further, lower reported affective arousal was significantly associated with a greater propensity for errors at the ambiguous cues (λ_amb_, LRT = 4.697, p = 0.030), and there was a weak tendency for a lower reported affective arousal to be associated with a weaker bias towards the ‘safe’ response ($\beta_{0}^{\delta }$: LRT = 2.851, p = 0.091).

**Table 1:** Results of the statistical analysis of the estimates of parameter estimates.

Dependent Variable	Predictor Variable	LRT	p-Value	Significance
*B*	arousal	0.006	0.939	
	valence	0.007	0.933	
$\beta_{0}^{\delta }$	arousal	2.851	0.091	MNS
	valence	3.526	0.060	MNS
$\beta_{\bar{R}}^{\delta }$	arousal	0.069	0.792	
	valence	2.370	0.124	
$\beta_{O}^{\delta }$	arousal	0.713	0.399	
	valence	0.150	0.699	
λ_amb_	arousal	4.697	0.030	*
	valence	0.480	0.488	
λ_ref_	arousal	0.679	0.410	
	valence	0.021	0.885	
$\frac{\Sigma_{0}^{T}P_{t;\text{timeout}}}{t}$	arousal	<0.041	0.840	
	valence	6.662	0.010	*
*σ*	arousal	1.318	0.251	
	valence	0.110	0.741	

### 1.3 Influence of within-task experience on reported affect

**Fig 3:**
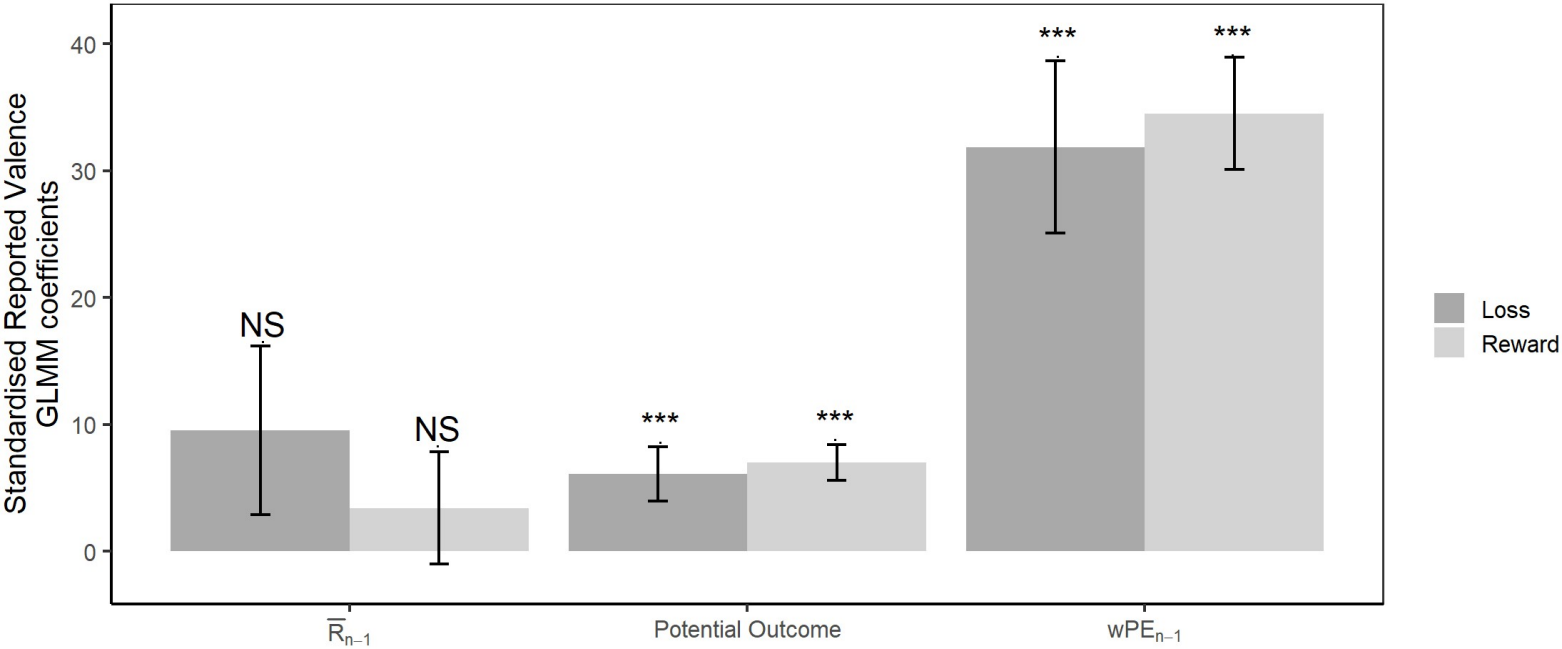
Valence GLMM coefficients. Standardised GLMM coefficients from the model of the reported affective valence for both the fluctuating reward and fluctuating loss condition. Positive values for the standardised GLMM coefficients reflect a positive relationship between reported valence and the predictor variable, while negative values reflect a negative relationship. Error bars represent one standard error.

Finally, we quantify model-agnostic effects of recent trial history on the reported affect. Participants reported significantly more positive affective valence when the weighted prediction error (*w*PE_*n*−1_; LRT = 66.411, p<0.001), and the potential outcome (*O*_*n*−1_; LRT = 27.132, p<0.001) were more positive (Fig 3). The average earning rate was not a significant predictor of reported affective valence (Fig 3; ${\bar{R}}_{n-1}$; LRT = 2.616, p = 0.106).

There was no difference in reported valence between the fluctuating reward and fluctuating loss conditions (LRT = 1.673, p = 0.196). Additionally, the interactions between the potential outcome and experimental condition (LRT = 0.124, p = 0.724), the weighted prediction error and condition (LRT = 0.113, p = 0.737), and also between the average earning rate and condition (LRT = 0.603, p = 0.437) were not significant (Fig 3).

**Fig 4:**
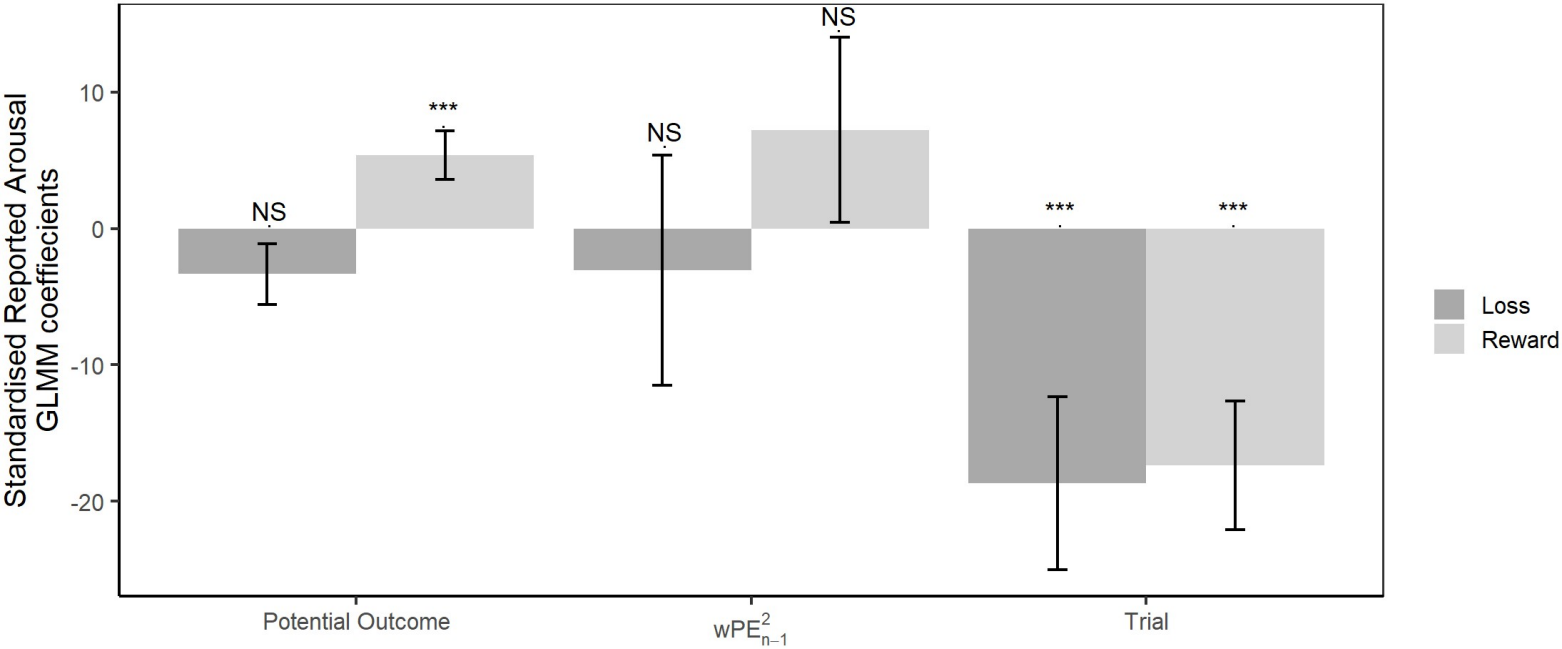
Arousal GLMM coefficients. Standardised GLMM coefficients from the model of reported affective arousal for both the fluctuating reward and fluctuating loss condition. Positive values for the standardised GLMM coefficients reflect a positive relationship between reported arousal and the predictor variable, while negative values reflect a negative relationship. Error bars represent one standard error.

Reported affective arousal and valence were not correlated in this study (LRT = 1.576, p = 0.209). The number of trials completed had a strong effect on reported arousal (LRT = 20.292, p<0.001) with participants reporting lower arousal when they had completed a greater number of trials. There were no significant effects of the squared weighted prediction error ($w{\text{PE}}_{n-1}^{2}$; LRT = 1.555, p = 0.212), potential outcome (LRT = 0.918, p = 0.338), and experimental condition (LRT = 0.011, p = 0.915) on reported arousal. However, there was a significant interaction between the potential outcome and experimental condition (Fig 4: LRT = 9.305, p = 0.002). Post-hoc analysis revealed that participants reported greater arousal when the potential outcome was more positive in the fluctuating reward condition (LRT = 9.027, p = 0.005), but this was not the case in the fluctuating loss condition (LRT = 2.284, p = 0.131). The interaction between condition and the squared weighted prediction error (LRT = 0.920, p = 0.337) was not significant (Fig 4).

## 2 Discussion

Performance in decision-making tasks can be influenced by predispositions and biases that participants bring with them to the task, but also by experiences that occur during the task. Previous research has typically focused on just one aspect of reward and loss experience and affect or decision-making (e.g., solely looking at the effect of prediction error on affect; or solely looking at the relationship between affect and decision-making). Here we provide the first in-depth analysis of how within-task experience may influence behaviour in the judgement bias test which is designed to assess the relationship between affective state and decision-making under ambiguity. Our study provides new information on the interplay between affect, decisions, and reward and punishment experience during short (within-task) timescales. The approach taken has the potential to reveal variables and constructs that underlie decision-making which in turn could be used as new markers of affective state. This has implications for a range of other tasks that investigate links between affective state and decision-making using serial trials that provide rewarding or punishing outcomes.

We focused on how the most recent decision outcome, recent prediction errors (difference between actual and expected decision outcomes), recent squared prediction errors (magnitude of unpredictability of outcomes) and the average earning rate during a task influence both decision-making and self-reported affective state. To achieve this, we used a judgement bias task in which we manipulated reward and loss experience by systematically varying either the threatened loss or offered reward on a trial-by-trial basis. We recorded the decisions made to probe stimuli and regularly asked participants to report their affective state using an affect grid. Decision-making data were analysed using a model-dependent analysis which interpreted decision-making in the judgement bias task through the lens of a POMDP. This novel model of data from a judgement bias task allowed insight into the influence of reward and punisher experience on decision-making and reported affect.

We made a number of predictions about the relationship between reward and loss experience, affect, and decision-making, specifically that; an overall more negatively-valenced affective state would be associated with more risk-averse decision-making, both a lower average earning rate and more negative prediction errors would be associated with more negative-valenced affect and consequently more risk-averse decision-making. Although we found that more negative prediction errors were associated with more negatively-valenced affect, we found no evidence that affective valence was associated with the average earning rate, or that prediction errors influenced decision-making. Contrary to our predictions, we found that more positive affect tended to be associated with more risk-averse decision-making, and that a greater average earning rate was associated with more risk-averse decision-making. We also obtained several findings unrelated to our initial set of predictions.

Here we discuss our findings focusing first on within-task experience of reward and losses on decision-making. We then consider how these variables underlying decision-making relate to self-reported affect before discussing the links between within-task reward / loss experience and affect. We end by considering dissociations between effects of recent reward and loss experience on decision-making and self-reported affect.

### 2.1 Does recent experience of reward and loss modulate decision-making?

Our findings indicate that ongoing experience of rewards and losses is indeed a key determinant of decision-making within the judgement bias task. Specifically, the average earning rate and previous outcome were important determinants of the participants’ discretised reaction times. Participants were more risk-averse when recent outcomes or the average earning rate were higher. A similar judgement bias task with rodents also found that a rewarding outcome on one trial resulted in a greater likelihood of making a risk-averse response on the subsequent trial [50]. Both findings contrast with our prediction that a greater average earning rate (and a better recent outcome) reflect the favourability of within-test experience and hence should be associated with more risky decision-making. Instead, within-task experience of a rewarding event or environment appears to increase the likelihood of risk-averse responses on subsequent trials. In each task, the ‘stay’ response could be considered both risky and exploratory as the outcome of the ‘stay’ action is variable while the outcome of the ‘go’ action is fixed. Accordingly, the relationship between decision-making and both the average earning rate and previous outcomes would be consistent with previous findings from animal studies showing that risk and exploration can become greater as overall conditions become less favourable [51, 52], a trend that may be explained by individuals in poorer conditions having ‘little to lose’. Why this appears to be more evident in a short-time window (within-task) compared to a longer-time window when less favourable pre-task conditions are often associated with less risky within-task decision-making [15] requires further investigation.

### 2.2 How do variables underlying decision-making behaviour relate to self-reported affect?

We found that baseline biases were related to self-reported affective valence; individuals who were more risk-averse (according to the model bias parameter) tended to overall report more positive affect. This result is reminiscent of cautious optimism: the finding that positive affect can induce greater caution, despite a more optimistic belief about the outcome of decisions [53–55]. Cautious optimism has been explained as a self-protecting mechanism which leads individuals to make decisions that allow them to maintain their positive affect [53–55]. Therefore, this finding may reflect that individuals in more positive affective states were more averse to losing money because it might have threatened their positive affective state. Indeed, this explanation is consistent with our finding that a more favourable within-test experience increases risk-aversion.

In contrast to these findings, negative affect is more typically associated with greater risk-aversion/‘pessimism’ [14, 15, 17]. However, our study was conducted over a short-time scale in a non-clinical population, and differences between findings may thus reflect differences in the effect of short- as opposed to longer-term (and clinical) negative affect on decision-making.

Reported valence was also associated with an aspect of decision-making unrelated to ‘optimistic’/‘pessimistic’ biases. Specifically, participants who reported more negative affective valence were found to have poorer time estimation, as characterised by the model timeout probabilities. It could be that greater uncertainty about the time remaining on the trial increases negative affect, given that uncertainty is often considered to be aversive (although we found no evidence that recent unpredictability within the task is aversive) [23, 56]. Alternatively, this result could reflect the possibility that negative affect induces poorer time estimation. This explanation would be supported by a number of studies that have found a relationship between mood and interval timing [57, 58], although it is important to note that evidence regarding the relationship between affect and time perception is conflicting [58, 59]. Moreover, the dopaminergic system has been implicated in both time perception [60] and mood disorders [61, 62], providing a potential neurobiological basis for this finding.

Affective arousal was also associated with aspects of decision-making both related and unrelated to ‘optimistic’/‘pessimistic’ biases. Firstly, there was a tendency for greater arousal to be associated with greater risk-seeking. Hence, high-arousal negative-valence (i.e., anxiety-like) affective states tended to be associated with greater risk-seeking, while low-arousal, positive-valence (i.e., calm-like) states were associated with greater risk-aversion. Although anxiety-like states are typically hypothesised to induce risk-aversion/‘pessimistic’ decision-making, both human [63] and rodent [64] studies have demonstrated that chronic stress can induce risky decision-making.

Secondly, lower arousal was associated with a higher frequency of stimulus-independent errors when presented with the ambiguous cues. This result is fairly intuitive; lower engagement with the task and poorer concentration, resulting from lower arousal, should increase an individual’s propensity for errors [65, 66].

### 2.3 Does recent experience of reward and loss modulate self-reported affect?

Our results corroborate the finding that affect reflects relative levels of rewards [21, 34]. A more positive weighted prediction error led to more positive reported affective valence, indicating that positively valenced affect arises in environments where rewards are greater than expected. However, we found no evidence that the average earning rate (i.e., a measure of absolute reward and loss experience) within the task modulated affective valence. This is at odds with the theoretical framework for affect proposed by Mendl et al. (2010) [25] in which affective valence is hypothesised to reflect environmental levels of rewards and punishers; high-reward environments are considered to induce a positively valenced state to drive reward acquisition, and high-punisher environments are suggested to induce a negatively valenced state to promote punisher avoidance (see also [16, 67, 68]). As this task was conducted over a short timescale, further research should investigate whether absolute and relative levels of rewards and punishers would indeed influence longer-term affect (mood) as opposed to transient trial-by-trial fluctuations in reported affect.

Participants also reported more positive affective valence when the most recent trial had offered a higher reward or lower loss, indicating that the opportunity to win greater amounts is likely to induce a positive emotional state, while the potential to lose greater amounts is likely to induce a negative emotional state [69].

Affective arousal was also influenced by experience during the task. Greater affective arousal was reported when the offered reward was higher in the fluctuating reward condition, suggesting that high stakes trials required greater alertness. Participants also reported lower arousal as the number of trials completed increased, likely indicative of a degree of boredom.

### 2.4 Dissociations between effects of recent experience of reward and loss on decision-making and self-reported affect

Recent prediction errors during the task influenced affective valence but not decision-making and judgement bias. Conversely, despite influencing decision-making, there was no evidence that the average earning rate or previous outcome influenced reported affect, although baseline biases in risk-aversion tended to be associated with both reported affective valence and arousal. These findings indicate that particular influences on decision-making within the judgement bias task need not also have an effect on self-reported affective valence, and vice versa. This raises the question of whether effects on decision-making of recent experience are necessarily mediated by changes in affective state. It is possible, for example, that transient fluctuations in affect (such as those associated with the prediction error) do not exert a strong influence on decision-making, while longer-term affect (i.e., mood) may be a more important determinant of decision-making. This might be compounded in the current study by the use of rapidly fluctuating offered rewards and threatened losses creating a relatively volatile environment which is less informative about the outcome of future decisions [70, 71].

These possibilities should be explored in future studies, especially as the general relationship between affect and ‘optimistic’ or ‘pessimistic’ decision-making is supported by a recent meta-analysis of animal judgement bias studies. Pharmacological and environmental manipulations of animal affect alter judgement bias as predicted [14, 15] but there is considerable heterogeneity of study findings and variation in task characteristics and within-task experience may be one reason for this. The influence of arousal on decision-making identified in this study, albeit weak, may also explain some of the heterogeneity observed across judgement bias studies.

### 2.5 The POMDP model

The POMDP model provided greater insight into decision-making, and the relationship between affect and decision-making, than simpler, statistical, analyses. The model predicted the discretised reaction times very well, and the choices predicted by the model compared favourably with an alternate model of judgement bias choices. The model generalises aspects of diffusion-to-bound like models in particular because of the risk associated with stochastic timing.

In contrast to typical psychometric function fitting, we included an additional lapse rate parameter to characterise the psychometric function in our POMDP model (which was justified according to both AIC and BIC scores), to allow for separate lapse rates for the ambiguous and reference stimuli. This reflects that the likelihood of errors differs between trials where there is little doubt about the direction of motion of the RDK, and trials where direction of motion is difficult to detect—which may arise from the application of a different strategy for each of these cases.

Importantly, the results of this study suggest that the latent variables underlying decision-making that are revealed by the model may provide a better measure of affect than judgement bias itself. In particular, variation in time estimation (characterised by *ζ* and *ϕ*) within the task may provide a measure of affective valence, while propensity for errors (characterised by λ_amb_) may provide a measure of affective arousal. This possibility should be investigated in future studies.

In addition to assessing the external validity and reliability of these results, an important next step to assess our novel POMDP model will be to investigate how the current model parameters relate to neurobiological processes. Endogenous fluctuations in dopaminergic activity in the midbrain have been shown to correspond to within-subject variability in risky decision-making [72]; hence we might expect our results to correspond with the mean or standard deviation of these fluctuations within an individual participant. Likewise, dopaminergic activity might underlie the relationship between variability in time estimation and reported affective valence [60].

## 3 Conclusions

Serial decision-making tasks have been used to study and measure affective states or disorders. However, little consideration has been given to the potential influence of outcomes within these tasks on subsequent decision-making and affect. We hypothesised that a more favourable within-task experience would lead to more positively-valenced affect and riskier decision-making. This study revealed a number of novel relationships between within-task reward experience, affective state, and behaviour in a decision-making task—some of which were in direct contrast to our hypotheses. In line with our hypotheses and previous research, we found that individuals reported more positive affective valence during epochs of the task when recent prediction errors were more positive, and offered outcomes were more positive. The study also highlighted the role of the favourability of task outcomes in modulating risk-aversion; contrary to our hypotheses, increased risk-averse decision-making was observed when the average earning rate and most recent outcome were higher. Finally, in contrast to our hypotheses, but in line with the aforementioned findings, increased risk-aversion tended to be associated with high-arousal and negative-valence affective states. Thus, within-task experience influenced both decision-making and affect, and this should be considered when using serial decision-making tasks to investigate affect.

The findings, which linked reward and punisher experience and decision-making, would not have been revealed without the development of a novel computational model for examining decision-making in the judgement bias task. Overall, our results provide evidence that within-task rewarding and punishing experiences determine both future decision-making and subjectively experienced affective state. This has implications for other tasks that are employed to detect links between serial decision-making and affective states or disorders, and highlights the potential for computational modelling to reveal novel variables and constructs underlying within-task decisions that can be used as new markers of these states. In future studies, it would be worthwhile to attempt to replicate these results, and to investigate aberrant decision-making in clinical populations using the methodology outlined here.

## 4 Methods

### 4.1 Ethics statement

Participants provided written, informed consent, and the study was approved by the Faculty of Science Research Ethics Committee at the University of Bristol.

### 4.2 Participants

Thirty-nine students from the University of Bristol participated in the study and were paid £5 per session for their participation plus a performance-dependent bonus.

### 4.3 Procedure

Using an independent-subjects design, 20 participants undertook the experiment in the context of obtaining variable rewards (*fluctuating reward condition*), and 19 in the context of avoiding variable losses (*fluctuating loss condition*). The task was written in MATLAB (MathWorks, Natwick, MA, USA) using the PsychToolBox package [73].

### 4.4 Judgement bias task

The judgement bias task is a widely-used paradigm to investigate putative affect and decision-making in non-human animals [14, 15]; in particular it assesses whether an animal makes a risky (‘optimistic’) or safe (‘pessimistic’) action when sensory information about the outcome of the risky action is ambiguous. Our task was a human version of the automated rat judgement bias task described by Jones et al. (2018) [74]. In their task, rats self-initiated trials by putting their snout in a trough. This led to the presentation of a tone, the frequency of which provided more or less ambiguous information about the outcome of the risky action (‘stay’; where the rat kept their snout in the trough). The rats had two seconds, during which the tone was played, to decide whether to make this risky action or whether to make the safe action (‘go’: in which the rat removed their snout from the trough) (see [75] for discussion of Pavlovian influences in this task).

Here, we replace the auditory stimuli with random dot kinomatograms (RDKs) which are widely-used sensory stimuli for primates (including humans). The RDKs comprised 100 dots which moved at a speed of 780 pixels per second displayed within a circular aperture with a diameter of 208 pixels. The dots were square with a width and length of 3 pixels. Signal dots (i.e., those moving in the same direction) were selected at random on each frame, and the remaining dots moved in a random direction on each frame. Any dot that went outside of aperture was replotted in a random location within the aperture on the subsequent frame. The direction of motion of the signal dots was always either leftwards or rightwards, and the proportion of dots moving coherently (i.e., the number of signal dots; termed the ‘coherence level’) was varied across trials to alter the difficulty with which the motion could be classified by the participant as leftwards or rightwards. In all blocks of trials, the signal direction of motion was equally often leftwards and rightwards, and each coherence level used within each block occurred an equal number of times within each direction of motion. The order of trials was randomised.

Instead of initiating trials by placing their nose in a trough, the human participants were instructed to initiate trials by pressing and holding the ‘enter’ key. The ‘risky’ action was to continue pressing the key for the two seconds of the RDK presentation (‘stay’), while the ‘safe’ action was to release the key (‘leave’). Execution of the ‘stay’ response was the ‘risky’/‘optimistic’ decision, as it could result in either a reward or loss, while execution of the ‘go’ response was the ‘safe’/‘pessimistic’ decision, as it resulted in neither a reward nor loss. Half of the participants were told that when the direction of motion was rightwards (threatened loss trials), they must release the ‘enter’ key (‘go’) prior to two seconds to avoid a loss (see below), and when the motion was leftwards (offered reward trials) they must continue to press the ‘enter’ key (‘stay’) for two seconds to obtain a monetary reward (see below), while the other half of the participants were told the obverse (i.e., leftwards = threatened loss; rightwards = offered reward). The semantics of ‘stay’ and ‘go’ were the same across the groups.

**Fig 5:**
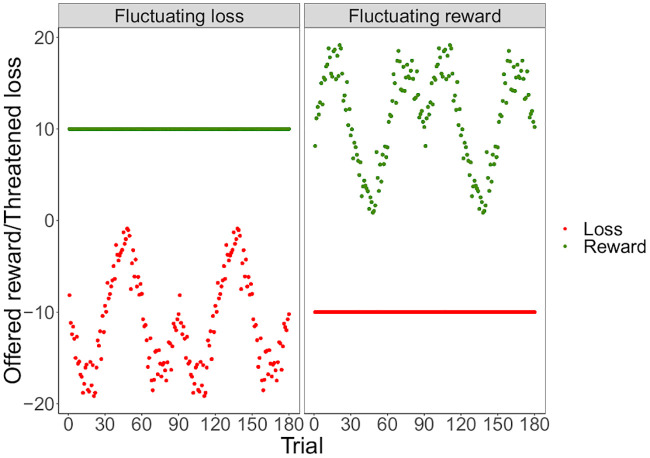
Offered rewards and threatened losses. Offered monetary reward and threatened monetary loss on each trial of the judgement bias task in the fluctuating reward and loss conditions.

Following each presentation of the RDK and response, participants were shown on-screen feedback. For the ‘offered reward’ trials, the correct response was to continue holding the key for two seconds (‘stay’) to gain a monetary reward, while for the ‘threatened loss’ trials the correct response was to release the key prior to two seconds (‘go’) to avoid a monetary loss. In the first two training blocks either ‘Correct’ (in green font) or ‘Incorrect’ (in red font) was displayed for 1.5s. In the final training block and test block, participants could win or lose money according their responses. In the final training block, they were informed that they would win or lose a multiple of £10, otherwise they would get £0. For the test block, following [37] and [38], the notional amount rewarded in the fluctuating reward condition, or potential loss, in the fluctuating loss condition, for correct or incorrect responses respectively varied across trials according to a sine function with added noise. In the fluctuating reward condition, the rewards shown to the participant varied between £0.87 and £19.16 and the loss was fixed at £10. In the fluctuating loss condition, the losses shown to the participant varied between £0.87 and £19.16 and the reward was fixed at £10 (Fig 5). The participant was truthfully informed that they would receive an amount proportional to their total wins and losses. To illustrate these amounts as clearly as possible, participants were informed about these values using connected green and red bars (with lengths proportional to the potential reward and loss respectively, and amounts written in figures at their ends) shown on the screen for 1.5s prior to each trial (see Fig 1). The amount won or lost was displayed on screen for 1.5s following each trial; this figure was green when a correct response was made and red otherwise. Participants were told that the monetary amounts they saw on the feedback screens during the final training and test block (see Fig 1) would be multiplied by a factor, and then added to or deducted from an initial £2 endowment, and a £5 turn-up fee. To sustain motivation across the test session, participants were informed that the top-three ranking participants would have their bonus doubled.

The particulars of each trial on the human task are as follows (see S1 Video File for example, and see Fig 1): participants were first shown the potential monetary outcome of the ‘stay’ action on-screen (as described above) and following acknowledgement of this by pressing the ‘enter’ key were first instructed by an on-screen prompt to press and hold the ‘enter’ key. This led to a fixation cross being displayed for 500ms, followed by a RDK displayed for 2000ms. The duration of the RDK display was 2000ms regardless of choice. Participants were required to make one of two responses to RDKs; (1) continue to press the ‘enter’ key (‘stay’) or (2) release the ‘enter’ key (‘go’). The ‘stay’ response resulted in a reward if the RDK moved in the rewarded direction (either leftwards or rightwards), and a loss if the direction of the RDK was in the alternate direction. The magnitude of the reward or loss varied across trials in a systematic manner. The ‘go’ response resulted in neither a reward nor loss. The outcome of their action was then displayed on screen for 1.5s.

Participants completed three training blocks consisting of 24, 60, and 24 trials respectively, followed by one test block of 180 trials. In the first training block, the direction of motion of the dots were unambiguous, with a coherence level of 0.32 (i.e., 32/100 dots moving coherently in the same direction). In the second training block, the direction of motion of the dots was difficult for the participants to determine (i.e., ambiguous) on a third of trials with a coherence level of 0.04, and unambiguous on two thirds of trials with coherence levels of 0.32 or 0.16. Stimuli in the third training block and also in the test block had a coherence level of 0.16, 0.02, or 0.01, with ambiguous coherence levels (0.01 or 0.02) on two thirds of trials (see S1 Video File for examples). The coherence levels required for the direction of motion to be perceptually ambiguous and unambiguous were determined in a pilot study.

### 4.5 Self reports of affect

At the start of the test block of each task and following every 10 subsequent trials (see Fig 1), participants were asked to report their current mood using a 9 by 9 computerised self-report affect grid [39, 40]. To complete the affect grid, participants had to move a cross, which was initially central in the grid, to the location that best described their current mood using the arrow keys on a keyboard (see S1 Video File for example). Horizontal movements represented changes in mood valence, with movements to the right reporting a more positively valenced mood. Vertical movements represented arousal, with upwards movement reporting higher levels of arousal.

### 4.6 Model dependent analysis

#### 4.6.1 POMDP model

The judgement bias task is a partially observable Markov decision process (POMDP) by its very nature, since the stimulus that is presented can be ambiguous. Accordingly, the decision-making process was modelled as POMDP [76, 77] with a two-dimensional state space *s* = (*t*, *X*) in which participants accumulate evidence (*X*) from observations of the RDK (*x*) over (veridical) time (*t*). The true direction of motion of the RDK (*μ*) takes one of two values, 1 or −1. Here, *μ* = 1 represents motion in the favourable direction in response to which the participant should ‘stay’, to collect a reward; *μ* = −1 represents motion in the unfavourable direction in response to which the participant should ‘go’ before the 2*s* is up to avoid a loss. Participants have to use prior knowledge (such as the possible direction and coherence levels of the RDK) together with the evidence they collect (in a manner closely related to drift-diffusion modelling), and the costs and benefits of being correct or incorrect, to decide what to do. The participants’ capacity to perform interval timing of the stay period is noisy [78], making it hard for them to wait until the last possible moment in order to collect evidence about *μ*.

For convenience, we discretise the objective time between zero and two seconds into bins of Δ*t*, and use integer states *t* = {0, 1, 2, …, *T*}Δ*t*. Thus, we write *x*_*t*_ to represent the observations from time (*t* − 1)Δ*t* to *t*Δ*t*, and *x*_0:*t*_ to represent all the observations from the beginning of the trial up to time *t*. Given the coherence of the stimulus (written as *θ*), the participant’s relative belief that the stimulus is favourable at time *t* will depend on their prior belief that the stimulus would be favourable, the relative likelihood of their observations prior to time *t*, and the relative likelihood of the current observation:

$$\begin{array}{c} \text{log}\;(\frac{P(\mu \;=\;1|x_{0:t},\theta )}{P(\mu \;=\;-1|x_{0:t},\theta )})=\text{log}\;(\frac{P(\mu \;=\;1)}{P(\mu \;=\;-1)})+\text{log}\;(\frac{P(x_{0:t}|\mu \;=\;1,\theta )}{P(x_{0:t}|\mu \;=\;-1,\theta )})\\ =\text{log}\;(\frac{P(\mu \;=\;1)}{P(\mu \;=\;-1)})+\text{log}\;(\frac{P(x_{0:t-1}|\mu \;=\;1,\theta )}{P(x_{0:t-1}|\mu \;=\;-1,\theta )})+\text{log}\;(\frac{P(x_{t}|\mu \;=\;1,\theta )}{P(x_{t}|\mu \;=\;-1,\theta )}) \end{array}$$

We assume that the likelihood follows a Gaussian probability distribution with a mean dependent on the true value of *μ* and the coherence level *θ*, and (fixed) variance *σ*^2^ that reflects the participant’s ability to detect the direction of motion of the RDK:

$P\left(x_{t}|\mu ,\theta \right)∼N\left(x_{t}:\mu \theta ,\sigma^{2}\right)$. Thus, the relative posterior probability for the last sample is:

$$\begin{array}{cc} \text{log}\;(\frac{P(x_{t}|\mu \;=\;1,\theta )}{P(x_{t}|\mu \;=\;-1,\theta )}) & =\frac{2\theta }{\sigma^{2}}x_{t} \end{array}$$

the above equation can be rewritten as:

$$\begin{array}{c} \text{log}\;(\frac{P(\mu \;=\;1|x_{0:t},\theta )}{P(\mu \;=\;-1|x_{0:t},\theta )})=\text{log}\;(\frac{P(\mu \;=\;1)}{P(\mu \;=\;-1)})+\frac{2\theta }{\sigma^{2}}\sum_{\tau =0}^{t}x_{\tau } \end{array}$$

For convenience, we suppress the dependence of *μ* and *σ*^2^ on Δ*t*.

Again, for convenience, and since the participant is ignorant of *θ*, we consider the information state $X=\sum_{\tau =0}^{t}x_{\tau }$, accumulating statistics *X* in discrete steps *χ* × [⋯ −3, −2, −1, 0, 1, 2, 3, …] for discretisation *χ*:

The state space contains two special states along with *s* = (*t*, *X*): one, *s* = leave, is the inevitable consequence of choosing action GO; the other, *s* = timeout, arises after 2 seconds if the participant does not actively go.

We describe in stages the probabilistic transition structure of the chain, i.e., *T*_*s*;*s*′_(*a*), which is the probability of going from state *s* to state *s*′ when executing action *a*. First, we have *T*_*s*;leave_(GO) = 1, and *T*_*s*;leave_(*a*) = 0, *a* ≠ GO. Second, we simplify the stochastic, interval-timing [78] relationship between objective and subjective time by imagining that timeout can happen probabilistically when the participant chooses *a* = STAY. It would be more realistic for the participants’ time to evolve *subjectively* rather than *objectively*, and for timeout to happen deterministically in objective time. However, this would mean a non-uniform acquisition of evidence (i.e., the statistics of *x*_*t*_ would not be homogeneous), making for extra complexities of only modest import.

We therefore imagine that, from a participant’s perspective, the stay time is a random variable which is primarily determined by a gamma distribution Γ(*ζ*, *ϕ*), with shape and scale parameters *ζ* and *ϕ*, respectively. This then leads to a hazard function, which is defined as the probability of transitioning to timeout at each point in time assuming that the individual hasn’t already transitioned to timeout:

$$\begin{array}{c} P_{t;\text{timeout}}=P(\text{timeout}\in (t,t+1)\Delta t|\text{continuing}\;\text{at}\;t\;\&\;a_{t}=\text{STAY}) \end{array}$$

which can be calculated as

$$\begin{array}{c} P_{t;\text{timeout}}=\frac{\int_{t\Delta t}^{(t+1)\Delta t}d\tau \;\Gamma \left(\tau ;\zeta ,\phi \right)}{1-\int_{0}^{t\Delta t}d\tau \;\Gamma \left(\tau ;\zeta ,\phi \right)} \end{array}$$

However, we impose an actual time-out at the end of the trial by fiat:

$$\begin{array}{cc} P_{T;\text{timeout}} & =1 \end{array}$$

Next, if the participant knows the quality of the stimulus, *μ*, *θ*, and there is no timeout when choosing STAY, we have for a single sample of *x* over time Δ*t*:

$$\begin{array}{cc} P_{X;X^{\prime }}^{\mu ,\theta } & =P(x\in \chi \left(X^{\prime }-X\right)+\left[-\chi /2,\chi /2\right]|\text{no}\;\text{timeout},\text{STAY},\mu ,\theta )\\ & =[\Phi_{\sigma }(\chi \left(j-i\right)+\frac{1}{2}\chi -\mu \theta )-\Phi_{\sigma }(\chi \left(j-i\right)-\frac{1}{2}\chi -\mu \theta )] \end{array}$$

where Φ_*σ*_ is the cumulative normal function for $N(0,\sigma^{2})$, *j* maps the accumulated statistics represented by *X*′ to a specific location in the state space, and *i* maps the accumulated statistics represented by *X* to a specific location in the state space (i.e., *X* = *j* × *χ*).

**Fig 6:**
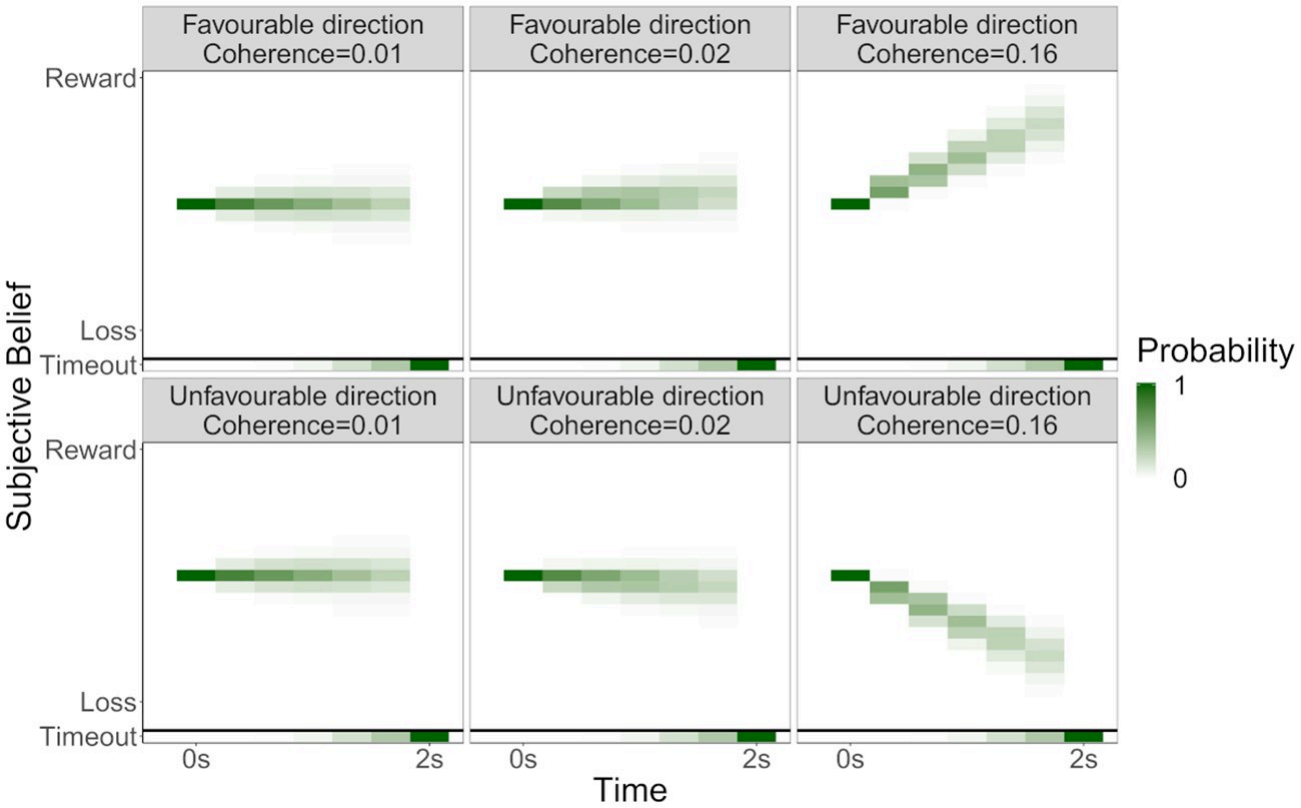
Transition probabilities. Heatmaps illustrating the probability that a participant transitions to each state given the stimulus (i.e., true values of *μ* and *θ*). The coherence level (*θ*) governs the rate of the transition towards a stronger belief that the ‘stay’ response will lead to a reward or loss; and the direction of the stimulus (*μ*) governs the direction of the transitions.

Of course, the participant does not know the true value of *μ* or *θ*. Thus, the evidence component of the subjective transition matrix *T*_*s*;*s*′_(*a*) comes from averaging over the possible *μ* and *θ*, given the information available at the current state, i.e., using the posterior probability (Fig 6):

$$\begin{array}{cc} P(\mu ,\theta |t,X) & =\frac{P(\mu )P(\theta )P(X|\mu ,\theta ,t)}{\Sigma_{\mu^{\prime }\in \left\{-1,+1\right\}}\Sigma_{k}P\left(\mu^{\prime }\right)P\left(\theta \right)P\left(X|\mu^{\prime },\theta_{k},t\right)}\;,\text{given}\;\text{priors:}\\ P(\mu =1) & =P\left(\mu =-1\right)=\frac{1}{2}\\ \;P(\theta =0.01) & =P\left(\theta =0.02\right)=P\left(\theta =0.16\right)=\frac{1}{3} \end{array}$$

where *X* ∼ *N*(*μθt*, *tσ*^2^).

In sum, the subjective transition structure from current state *s* = (*t*, *X*) is therefore given by:

$$\begin{array}{cc} T_{(t,X);\text{leave}}\left(\text{go}\right) & =1\\ T_{(t,X);\text{timeout}}\left(\text{STAY}\right) & =P_{t;\text{timeout}}\\ T_{\left(t,X\right);\left(t+1,X^{\prime }\right)}\left(\text{STAY}\right) & =\left(1-P_{t;\text{timeout}}\right)(\sum_{\mu \in \{-1,+1\}}\sum_{k}P\left(\mu ,\theta_{k}|t,X\right)P_{X;X^{\prime }}^{\mu ,\theta_{k}})\;t<T \end{array}$$

A participant’s policy *π* is determined by the long-run values $Q_{L;R}^{\pi }\left(s,a\right)$ of executing action *a* in state *s* = (*t*, *X*), and then following the policy thereafter, where *R* and *L* are the potential reward and loss. In general, these action values are determined by the Bellman equation [79]. We assume that no discounting occurs given the short duration of the trial. In this case:

$$\begin{array}{cc} Q_{L;R}^{\pi }\left(s,\text{GO}\right) & =0\\ \;Q_{L;R}^{\pi }\left(s,\text{STAY}\right) & =T_{s;\text{timeout}}\left(\text{STAY}\right)V_{L;R}^{\text{timeout}}\left(s\right)+\sum_{s^{\prime }=\left(t+1,X^{\prime }\right)}T_{s;s^{\prime }}\left(\text{STAY}\right)V_{L;R}^{\pi }\left(s^{\prime }\right) \end{array}$$

where *s*′ = (*t* + 1, *X*′).

Here, the expected timeout value depends on the likelihood of the two possibilities:

$$\begin{array}{c} V_{L;R}^{\text{timeout}}\left(s\right)=(R\times P(\mu \;=\;1|s))+(L\times P(\mu \;=\;-1|s)) \end{array}$$

**Fig 7:**
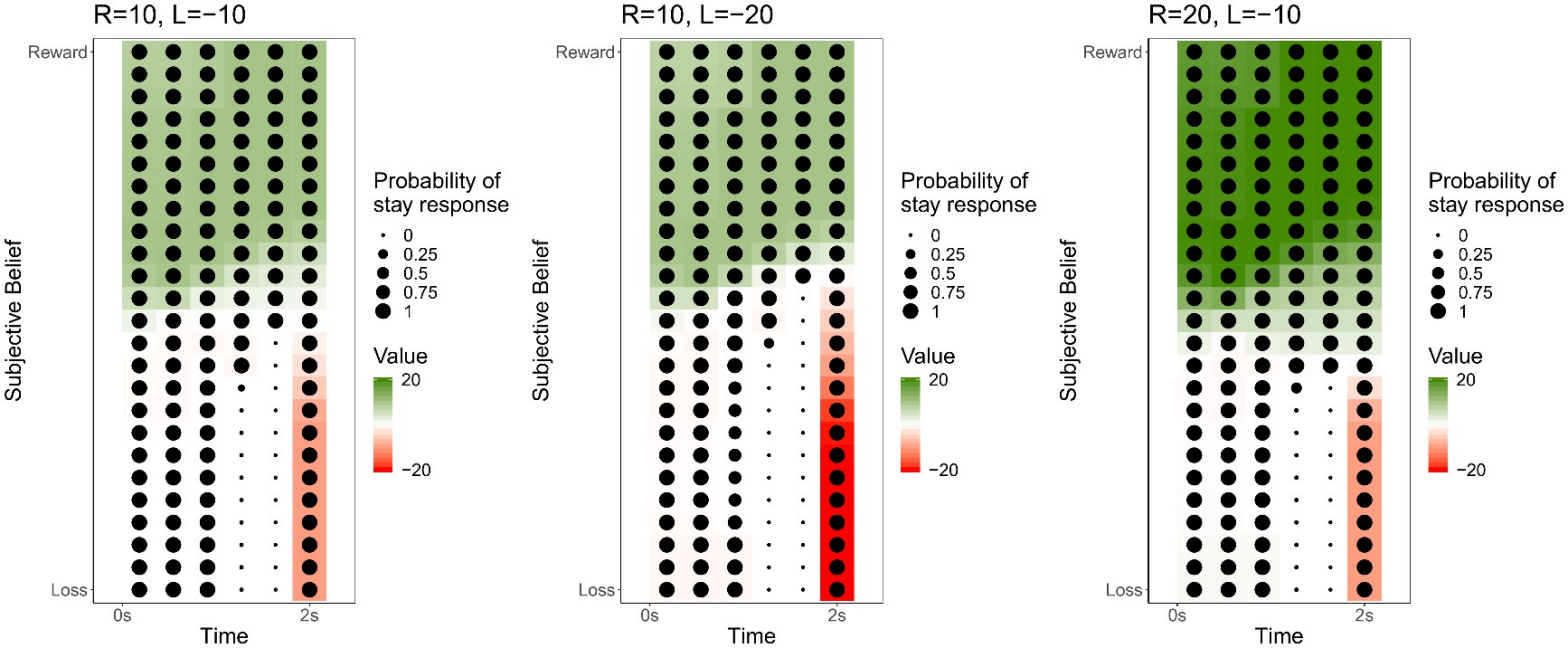
State values. Heatmap illustrating the value of occupying each state as the value of the offered reward and threatened loss varies. The state values earlier in the trial in which there is a weaker belief that the trial will be rewarded (i.e., bottom left quadrant of each heatmap), are close to zero; this reflects that the ‘go’ action is the most likely future action and hence the trial outcome will most likely be zero. The value of states representing no strong belief changes across time (i.e., middle third of the y-axis on each heatmap); this reflects that transitions to a state with a high value (e.g., resulting from a strong certainty of reward) may be considered probable earlier in time, but if there is uncertainty about the stimulus further into the trial, then it is most likely that the stimulus has a low coherence and that reaching a high value state is less probable. The overall value of states representing no strong belief about the stimulus are most strongly modulated by the subjective value of the reward and loss; being higher when the reward is higher, and lower when the loss is higher.

Further:

$$\begin{array}{c} V_{L;R}^{\pi }\left(s^{\prime }\right)=\pi_{L;R}\left(s^{\prime },{\text{GO}}^{\prime }\right)\times 0+\pi_{L;R}\left(s^{\prime },\text{STAY}\right)Q_{L;R}^{\pi }\left(s^{\prime },\text{STAY}\right) \end{array}$$

where the policy *π* is determined by:

$$\begin{array}{cc} \pi_{L;R}\left(s^{\prime },\text{STAY}\right) & =\frac{\lambda }{2}+\left(1-\lambda \right)\sigma \left(B\left[Q_{L;R}^{\pi }\left(s^{\prime },\text{STAY}\right)-Q_{L;R}^{\pi }\left(s^{\prime },\text{GO}\right)+\delta -n\beta_{n}\right]\right)\\ \pi_{L;R}\left(s^{\prime },{\text{GO}}^{\prime }\right) & =1-\pi_{L;R}\left(s^{\prime },\text{STAY}\right) \end{array}$$

with *σ*(*z*) = 1/(1 + exp(−*z*)) being the logistic sigmoid, λ (which could take one of two values: λ_amb_ for ambiguous stimuli and λ_ref_) and *B* being lapse and inverse temperature parameters respectively, *δ* characterising biases towards or away from the ‘stay’ (‘optimistic’) response (c.f. [44]), and *β*_*n*_ being a scale parameter to account for the effects of number of trials completed on decision-making (Fig 7). The latter was included as previous research has demonstrated that the number of trials completed can influence judgement bias [74].

To calculate the distribution of leaving times, we consider the probability, $P_{s}^{\text{remain}}\left(\mu_{n},\theta_{n},R_{n},L_{n},n\right)$, of staying until state *s* = (*t*, *X*) on trial *n*, which is defined by direction *μ* = *μ*_*n*_, coherence *θ* = *θ*_*n*_, reward *R* = *R*_*n*_ and loss *L* = *L*_*n*_. This enjoys a recursive form:

$$\begin{array}{cc} P_{0,X}^{\text{remain}}\left(\mu_{n},\theta_{n},R_{n},L_{n}\right) & =1\\ P_{t,X}^{\text{remain}}\left(\mu_{n},\theta_{n},R_{n},L_{n}\right) & =\int_{x_{t}}dx_{t}\;P\left(x_{t}|\mu_{n},\theta_{n}\right)\times \\ & \;P_{t-1,X-x_{t}}^{\text{remain}}\left(\mu_{n},\theta_{n},R_{n},L_{n}\right)\times \\ & \;\pi_{L;R}\left(\left(t-1,X-x_{t}\right),\text{STAY}\right)\left(1-P_{t-1;\text{timeout}}\right) \end{array}$$

from which we can calculate the overall probability of timing out (rather than leaving) as:

$$\begin{array}{cc} P^{\text{timeout}}\left(\mu_{n},\theta_{n},R_{n},L_{n}\right) & =\int_{X}dX\;\sum_{t=0}^{T}P_{t,X}^{\text{remain}}\left(\mu_{n},\theta_{n},R_{n},L_{n}\right)\pi_{R_{n},L_{n}}\left(\left(t,X\right),\text{STAY}\right)P_{t;\text{timeout}} \end{array}$$

As we hypothesise that judgement bias will depend on past experience, specifically the average earning rate (${\bar{R}}_{n-1}$), the weighted (low pass filtered—i.e., attenuated across trials) prediction error (*w*PE_*n*−1_), the weighted (low pass filtered—i.e., attenuated across trials) squared prediction error ($w{\text{PE}}_{n-1}^{2}$), and the most recent outcome (*O*_*n*−1_) we allow *δ* potentially to depend additively on these values as well as on a constant term, which reflects baseline individual variation in these parameters. To allow the relative contribution of ${\bar{R}}_{n-1}$, *w*PE_*n*−1_, $w{\text{PE}}_{n-1}^{2}$, *O*_*n*−1_ to *δ* to vary, we scale ${\bar{R}}_{n-1}$, *w*PE_*n*−1_, $w{\text{PE}}_{n-1}^{2}$, and *O*_*n*−1_ by weighting parameters:

$$\begin{array}{c} \begin{array}{cc} \delta & =\beta_{0}^{\delta }+\beta_{\bar{R}}^{\delta }{\bar{R}}_{n-1}+\beta_{w\text{PE}}^{\delta }w{\text{PE}}_{n-1}+\beta_{w{\text{PE}}^{2}}^{\delta }w{\text{PE}}_{n-1}^{2}+\beta_{O}^{\delta }O_{n-1} \end{array} \end{array}$$

${\bar{R}}_{n-1}$ and *w*PE_*n*−1_ are updated following the most recent outcome *O*_*n*−1_. The average earning rate reflects the learnt value of the test session from previous wins and losses and updates according to a Rescorla-Wagner learning model [80], with learning rate $\alpha_{{\bar{R}}_{n}}$:

$$\begin{array}{c} {\bar{R}}_{n}={\bar{R}}_{n-1}+\alpha_{\bar{R}}\left(O_{n}-{\bar{R}}_{n-1}\right) \end{array}$$

Further, following Rutledge et al. (2014) [21], the prediction error on past trials PE_1:*n*−1_ is weighted such that the influence of past prediction errors attenuates over trials. However, consistent with the average earning rate, the weighted reward prediction error (wPE) is a weighted average as opposed to a weighted summation as in [21]. The weighting is determined by forgetting factor *γ*_*w*PE_:

$$\begin{array}{c} w{\text{PE}}_{n}=\frac{1-\gamma_{w\text{PE}}}{1-\gamma_{w\text{PE}}^{n}}\Sigma_{i=1}^{n}\gamma_{w\text{PE}}^{n-i}{\text{PE}}_{i} \end{array}$$

Here, PE_*n*_ is defined as the difference between *O*_*n*_ and expected outcome of the trial prior to stimulus presentation, as given by the value function:

$$\begin{array}{c} {\text{PE}}_{n}=O_{n}-V_{R_{n},L_{n}}\left(\left(0,0\right)\right) \end{array}$$

In sum, the key parameters in the model were thus: *σ*, λ_amb_, and λ_ref_ (parameters characterising the psychometric function); *ζ* and *ϕ* (characterising the hazard function, and hence errors in time estimation); *B* (characterising decision stochasticity); $\beta_{0}^{\delta }$ (characterising a constant baseline decision-making biases); $\beta_{\bar{R}}^{\delta }$, $\beta_{O_{n-1}}^{\delta }$, $\beta_{w\text{PE}}^{\delta }$, $\beta_{w{\text{PE}}^{2}}^{\delta }$ (characterising the effect of the average earning rate, previous outcome, weighted prediction error, and squared weighted prediction error on decision-making biases); and *β*_*n*_ (characterising the effect of number of trials completed in the judgement bias task) (see Table 2). These parameters were not fitted simultaneously (see below section and Table 2).

**Table 2:** Glossary of parameters in the POMDP model of judgement bias, including the range of values that each parameter can take and the step at which they were added in stepwise model-fitting procedure.

Parameter	Range	Interpretation	Step
*α_R_*	[0, 1]	Learning rate for the average earning rate; higher values reflect more rapid updating of the average earning rate based on recent outcomes.	2
*B*	[0, ∞]	Inverse temperature parameter; higher values reflect lower decision stochasticity.	core
$\beta_{0}^{\delta }$	[−∞, ∞]	Baseline bias; higher values reflect an overall ‘optimistic’ bias	1
*β*_*n*_	[−∞, ∞]	Time-dependency of the policy: higher values reflect that the ‘stay’ action is less likely as more trials are completed.	2
$\beta_{O}^{\delta }$	[−∞, ∞]	Outcome dependent loss sensitivity; higher values reflect a greater ‘optimistic’ bias when the most recent outcome is higher.	2
$\beta_{\bar{R}}^{\delta }$	[−∞, ∞]	Average earning rate dependent bias; higher values reflect a greater ‘optimistic’ bias when the average earning rate is higher.	2
$\beta_{w\text{PE}}^{\delta }$	[−∞, ∞]	Weighted prediction error dependent loss sensitivity; higher values reflect a greater ‘optimistic’ bias when the weighted prediction error is higher.	2
$\beta_{w{\text{PE}}^{2}}^{\delta }$	[−∞, ∞]	Squared weighted prediction error dependent loss sensitivity; higher values reflect a greater ‘optimistic’ bias when the squared weighted prediction error is higher.	2
*γ*_*w*PE_	[0, 1]	Forgetting factor for the weighted prediction error; higher values reflect slower forgetting of previous prediction errors.	2
λ_amb_	[0, 1]	Lapse rate at the ambiguous stimuli: higher values reflect a greater likelihood of executing the action with the lowest value (i.e., the ‘wrong’ action).	core
λ_ref_	[0, 1]	Lapse rate at the reference stimuli: higher values reflect a greater likelihood of executing the action with the lowest value (i.e., the ‘wrong’ action).	core
*σ*	[0, ∞]	Slope parameter: higher values reflect a poorer ability to detect the true direction of the stimulus.	core
*ζ* and *ϕ*	[0, ∞]	Shape and scale parameters for the Gamma distribution that determines the timeout probabilities.	core

#### 4.6.2 Model-fitting

We fitted the choice/reaction time data (i.e., the probability of remaining until time t for ‘go’ responses; the probability of timeout for ‘stay’ responses) to a given model by maximum likelihood with multiple starting values, implemented using the fmincon and GlobalSearch functions in MATLAB. We added parameters in stepwise manner, and compared models using Bayes’s information criterion (BIC) values, and the final set of models was compared using both Aikake and Bayes’s information criterion (AIC and BIC) values (see S1 Appendix). Thus, parameters that did not increase the model parsimony were excluded. Parameters that characterised decision-making in the absence of biases (i.e., *B*, λ_amb_, λ_ref_, *σ*, *ζ*, and *ϕ*) were included in all models to account for their potential influence on behaviour (‘core’ parameters, Table 2). We first assessed whether the parameter that characterised constant biases in decision-making improved model parsimony (Step 1 parameter, Table 2); then the second set of parameters included in the model-fitting procedure were those that allowed within-task variation either resulting from experience or time (i.e., $\beta_{\bar{R}}^{\delta }$, $\beta_{w\text{PE}}^{\delta }$, $\beta_{w{\text{PE}}^{2}}^{\delta }$, $\beta_{O}^{\delta }$, *β*_*n*_, $\beta_{\bar{R}}^{\delta }$ & $\alpha_{\bar{R}}$, $\beta_{w\text{PE}}^{\delta }$ & *γ*_*w*PE_, $\beta_{w{\text{PE}}^{2}}^{\delta }$ & *γ*_*w*PE_) (Step 2 parameters, Table 2); and the final set combined the best models as determined through the previous steps. It was not feasible to fit all possible models to the data due to the computational complexity of the model-fitting procedure and the large number of possible combinations of model parameters. The model was found to provide accurate recovery of parameters, as determined by simulating data and assessing the correlations between the parameters recovered by the model and those used to simulate the data (see S1 Appendix). The code for the model was written in MATLAB (MathWorks, Natwick, MA, USA) and model-fitting was conducted using the Advanced Computing Research Centre High Powered Computing Facility at the University of Bristol. We used permutation tests to assess whether the parameter estimates from the most parsimonious model, as determined through model-fitting, differed significantly from zero.

Due to significant correlations between the weighted prediction error (*w*PE_*n*−1_), squared weighted prediction error ($w{\text{PE}}_{n-1}^{2}$), and previous outcome (*O*_*n*−1_), and likewise between the average earning rate ($\bar{R}$) and number of trials completed (*n*), with the extent of the correlation dependent on the model parameters, parameters characterising the influence of these variables on the same aspect of the decision-making process were not fitted simultaneously in the same model, but instead fitted separately and the goodness of fit of each model compared using their BIC values.

### 4.7 Statistical analysis: Correlation between parameter estimates and reported affect

To examine whether the parameter estimates correlated with reported affect, a general linear model was fitted to the parameter estimates with mean reported arousal and mean reported valence (from each individual’s affect grid data averaged across the session) as the predictor variables, as well as condition (i.e., fluctuating reward or fluctuating loss) as a control variable.

### 4.8 Statistical analysis: Influence of within-task experience on reported affect

Generalised linear mixed models (GLMMs) were fitted to the affect grid data (i.e., the x and y coordinates for the selected location on the affect grid across the test session) in R [81] using the nlme [82] package. Likelihood ratio tests were then used to assess whether the difference in model deviance was significant following removal of a parameter from a model.

Each GLMM included a random effect of participant and fixed effects of the potential outcome ($\frac{R_{n}+L_{n}}{2}$), and condition (i.e., fluctuating reward or fluctuating loss). To examine if and how affect depended on reward and punisher experience, the values of the average earning rate (${\bar{R}}_{n-1}$), weighted prediction error (*w*PE_*n*−1_), squared weighted prediction error ($w{\text{PE}}_{n-1}^{2}$), and previous outcome (*O*_*n*−1_) immediately prior to presentation of each affect grid were calculated (using the best-fit model with the parameter estimates for each individual, and standardised by dividing by the standard deviation for each individual), as well as number of trials completed, and included as predictor variables in the GLMMs of reported valence and reported arousal. Due to significant correlations between the weighted prediction error (*w*PE_*n*−1_), squared weighted prediction error ($w{\text{PE}}_{n-1}^{2}$), and previous outcome (*O*_*n*−1_), and also between the number of trials completed and the average earning rate (${\bar{R}}_{n-1}$), these variables were not included in the same GLMM but instead separate GLMMs including each variable were compared according to their BIC value and the GLMM which provided the best fit was selected for further analysis. We assessed the relationship between reported affective arousal and valence by fitting reported affective valence to reported affective arousal using an additional GLMM with the described random effect structure.

The GLMMs predicting affect also included interaction terms between each of the variables encompassing reward and punisher experience and condition. To investigate significant and marginally non-significant interaction terms, the data were split by condition and further GLMMs were fitted to these subsetted data. Post-hoc adjustment of p-values from these GLMMS was conducted using the false discovery rate.

## Supporting information

S1 Appendix Supplementary information about the POMDP model. (PDF)

S1 Data Raw experimental data from the judgement bias task. (CSV)

S1 Video File Shortened task demonstration video. (MOV)
