# Supplementary material for: Dissecting the links between reward and loss, decision-making, and self-reported affect using a computational approach
Source: PLoS Comput Biol. 2021 Jan 8;17(1):e1008555. doi: 10.1371/journal.pcbi.1008555 (PMC7819615; doi:10.1371/journal.pcbi.1008555)
Supplement: S1 Appendix — (PDF) [file pcbi.1008555.s001.pdf]

S1 Appendix
Supplementary information about the POMDP model: Dissecting the links between reward and loss,decision-making, and self-reported affect using a computational approach

Vikki Neville[1*], Peter Dayan[2◑], Iain D. Gilchrist[3], Elizabeth S. Paul[1◑], Michael Mendl[1◑],

1 Centre for Behavioural Biology, School of Veterinary Science, University of Bristol, Langford, United Kingdom
2 Max Planck Institute for Biological Cybernetics, Tübingen, Germany
3 School of Psychological Science, University of Bristol, Bristol, United Kingdom

◑These authors contributed equally to this work.
* vikki.neville@bristol.ac.uk

1 Parameter Recovery

To assess whether the model-fitting procedure could recover the true parameter values, we first generated sets of data using a wide range of sensible parameter values for each parameter and fitted the model to these data. To determine whether the model could reliably detect individual differences, we examined the extent to which the true and recovered parameters were correlated using Spearman's correlation coefficient. Parameter recovery, as assessed using Spearman's correlation coefficient (Table 1) and visual inspection of scatterplots (Fig 1), was very good.

S1 Table 1. Results of the correlation analyses of the true and recovered parameter values for all parameters

Parameter	Spearman's correlation coefficient	p-Value
$\alpha_{\bar{R}}$	0.915	<0.001
B	0.939	<0.001
β_0^δ	0.909	<0.001
β_n	0.903	<0.001
β_O^δ	0.762	<0.001
$\beta_{\bar{R}}^\delta$	0.847	<0.001
$\beta_{w\text{PE}}^\delta$	0.936	<0.001
$\beta_{w\text{PE}^2}^\delta$	0.888	<0.001
$\gamma_{w\text{PE}}$	0.946	<0.001
λ_{amb}	0.905	<0.001
λ_{ref}	0.902	<0.001
$P_{1;\texttt{timeout}}$	0.984	<0.001
$P_{2;\texttt{timeout}}$	0.984	<0.001
$P_{3;\texttt{timeout}}$	0.974	<0.001
$P_{4;\texttt{timeout}}$	0.944	<0.001
σ	0.986	<0.001

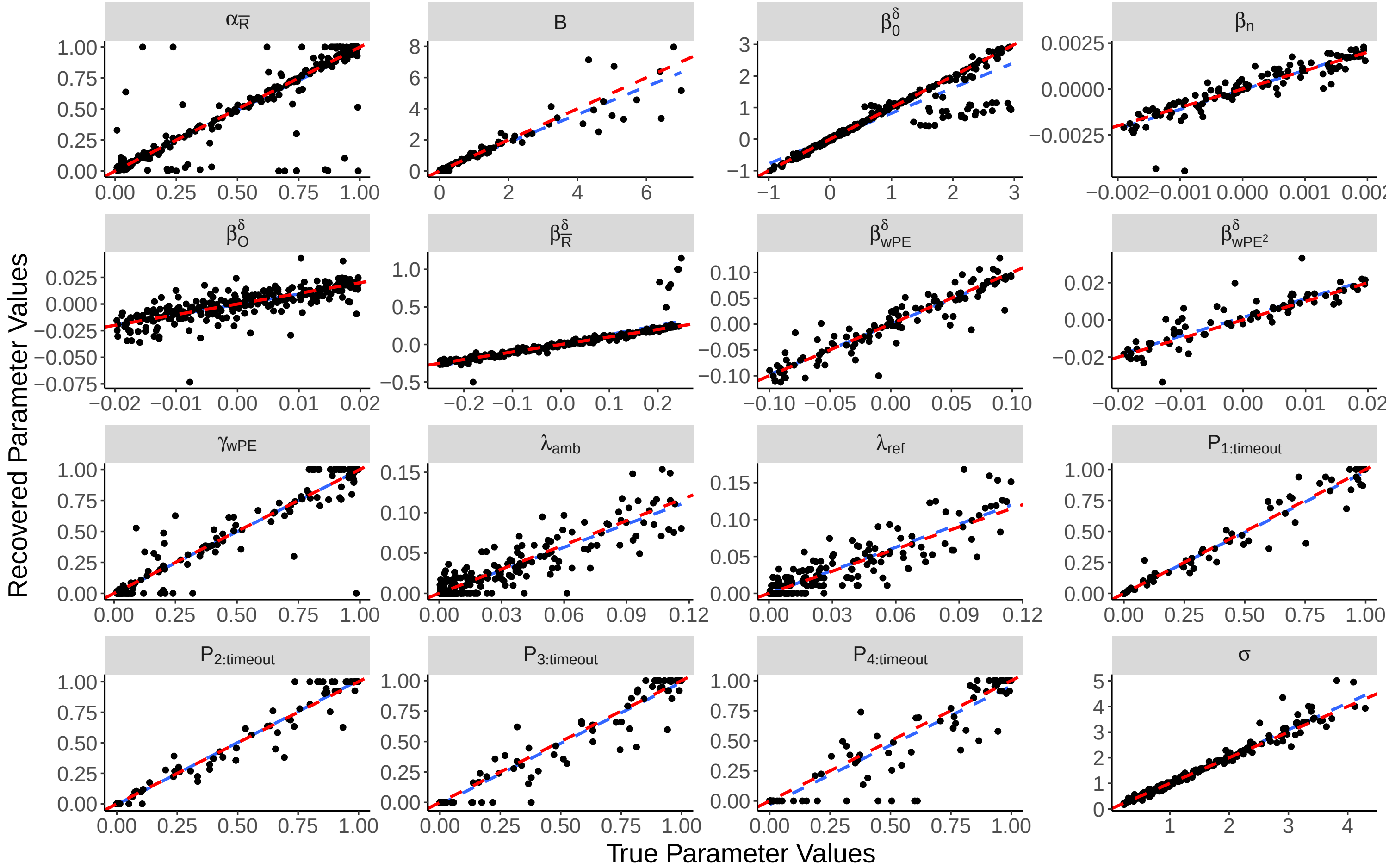

S1 Fig 1. Scatterplot of true and recovered parameter values for each parameter. The red dashed line is the line of equality, and the blue dashed line is the regression line

We then assessed the ability of the model-fitting procedure to recover the true parameter values, which included the range of those estimated by the best-fitting model of the observed data, when these parameters were fitted simultaneously. We excluded parameter estimates where optimisation had clearly failed (e.g. where there were extreme outliers across all parameter estimates). For the majority of parameters, there was a strong and significant correlation between the true and recovered parameters (Table 2), and the true value was close to the recovered value (see regression line vs. line of equality; Fig 2). However, parameter recovery, although still reasonable, was noticeably poorer for B, $\beta^{\delta}_{\bar{R}}$, and σ - in future studies, increasing the number of datapoints may improve the recovery of these parameters. Overall, we considered the parameters values obtained from the best-fitting model to be reliable estimates of the true parameter values.

S1 Table 2. Results of the statistical analyses of the true and recovered parameter values for parameters in the final model

**Parameter**	**Spearman's correlation coefficient**	**p-Value**
B	0.278	0.001
β^{δ}_{0}	0.688	<0.001
$\beta^{\delta}_{\bar{R}}$	0.216	0.019
β^{δ}_{O}	0.809	<0.001
λ_{amb}	0.882	<0.001
λ_{ref}	0.564	<0.001
$P_{1;\mathtt{timeout}}$	0.989	<0.001
$P_{2;\mathtt{timeout}}$	0.986	<0.001
$P_{3;\mathtt{timeout}}$	0.966	<0.001
$P_{4;\mathtt{timeout}}$	0.893	<0.001
σ	0.408	0.001

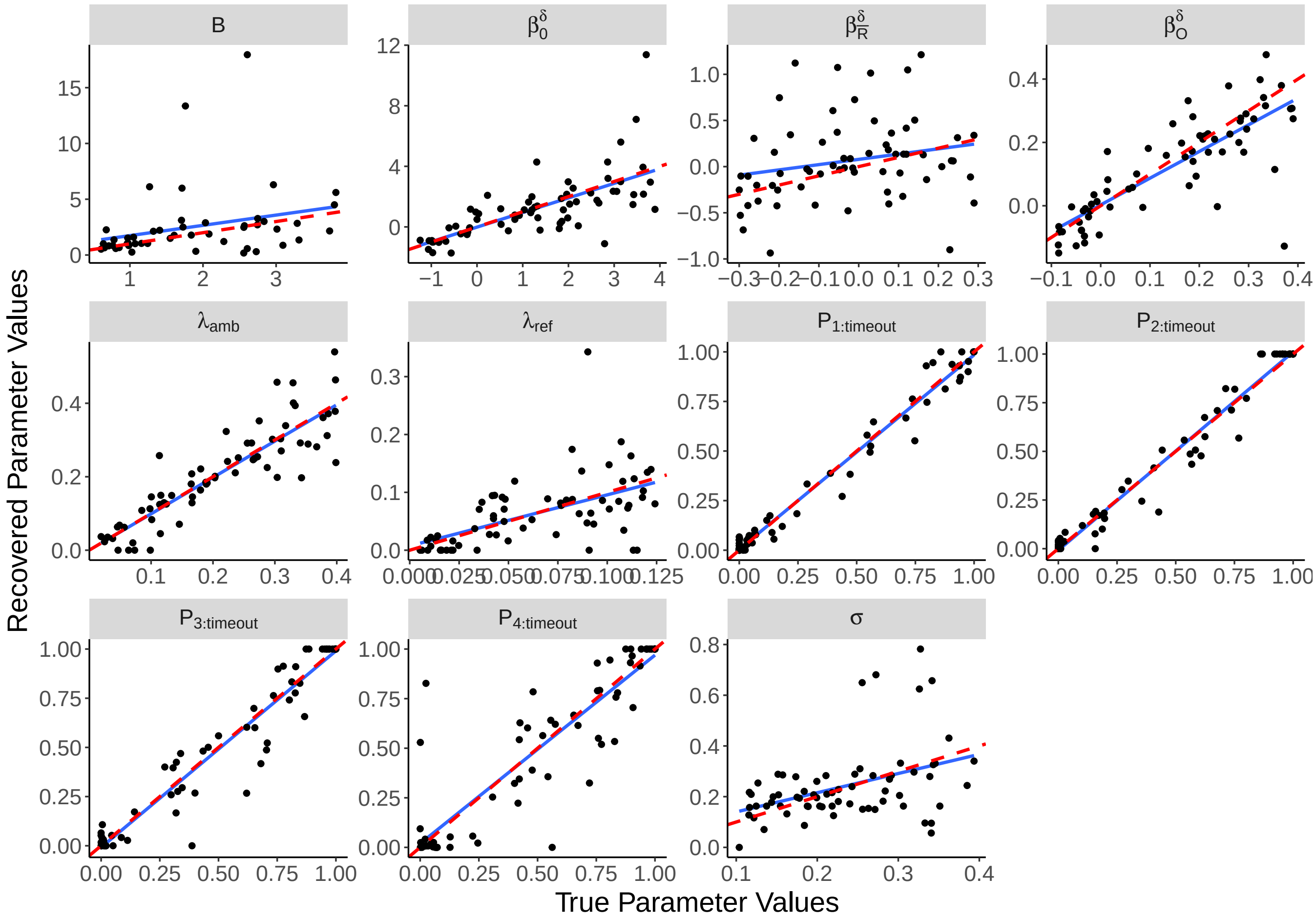

S1 Fig 2. Scatterplot of true and recovered parameter values for each parameter in the best-fitting model. The red dashed line is the line of equality, and the blue dashed line is the regression line

2 Model comparison

A number of models were fitted the data; Table 3 details these models and several measures of their relative goodness of fit. In a few instances, the optimisation procedure, despite using the GlobalSearch function which reported that the solver succeeded in all cases, did not identify a global minimum and produced a lower log-likelihood when an additional parameter was fitted to a participant's data compared to the model where this parameter was not fitted (i.e. a lower likelihood despite a greater number of parameters). Where this was the case, we instead used the log-likelihood and parameters from the model without the additional parameter, and penalised appropriately for the number of parameters (i.e. adding '2' to the AIC, or 'log(180)' to the BIC, for each case) and selection of the models where optimisation had failed (i.e. adding log(number of participant's data for whom a global minimum was not reached) to the AIC and BIC). At each step of fitting in our stepwise procedure, models were compared according to their BIC value. Then, the final set of models were compared according to their AIC and BIC values (both prior to and following adjustment). The AIC-best model following this adjustment for optimisation failure, and AIC-best and BIC-best model prior to this adjustment included nine parameters: σ, λ_{ref}, λ_{amb}, B, β_0^δ, $\beta_{\bar{R}}^\delta$, β_O^δ, ζ, and ϕ, and the BIC-best model post-adjustment included all the parameters listed except β_O^δ. As the estimates of β_O^δ, albeit very small, were found to differ significantly from zero according to a permutation test and also an alternative data analysis (see next section), and as the estimates of the same parameters from these two different best models were strongly correlated (i.e. correlation coefficients > 0.85), we selected the nine-parameter model as our final model. To ensure that this final model was appropriate, especially given the optimisation issues, we compared this model with two alternate models of judgement bias (see next section).

S1 Table 3. AIC and BIC values (both prior to and following adjustment for optimisation failures) for all models. Emboldened and underlined values indicate the lowest value (i.e. best-fitting model) for each measure of goodness of fit.)

**Model Parameters**	**adjusted AIC**	**adjusted BIC**	**AIC**	**BIC**
σ, λ_{amb}, λ_{ref}, ζ, ϕ, B	16809	17556	16809	17556
σ, λ_{amb}, λ_{ref}, β_0^δ, ζ, ϕ, B	15803	16674	141105	141977
σ, λ_{amb}, λ_{ref}, β_0^δ, $\beta_{\bar{R}}^\delta$, ζ, ϕ, B	15634	<u>**16631**</u>	59134	60131
σ, λ_{amb}, λ_{ref}, β_0^δ, $\beta_{\bar{R}}^\delta$, $\alpha_{\bar{R}}$, ζ, ϕ, B	15538	16659	272798	273918
σ, λ_{amb}, λ_{ref}, β_0^δ, $\beta_{w\text{PE}}^\delta$, ζ, ϕ, B	15677	16674	57840	58836
σ, λ_{amb}, λ_{ref}, β_0^δ, $\beta_{w\text{PE}}^\delta$, $\gamma_{w\text{PE}}$, ζ, ϕ, B	15647	16768	91896	93017
σ, λ_{amb}, λ_{ref}, β_0^δ, $\beta_{w\text{PE}^2}^\delta$, ζ, ϕ, B	15700	16696	15896	16892
σ, λ_{amb}, λ_{ref}, β_0^δ, $\beta_{w\text{PE}^2}^\delta$, $\gamma_{w\text{PE}}$, ζ, ϕ, B	15660	16780	80031	81152
σ, λ_{amb}, λ_{ref}, β_0^δ, β_O^δ, ζ, ϕ, B	15679	16675	95870	96866
σ, λ_{amb}, λ_{ref}, β_0^δ, β_n, ζ, ϕ, B	15677	16673	16022	17018
σ, λ_{amb}, λ_{ref}, β_0^δ, $\beta_{\bar{R}}^\delta$, $\beta_{w\text{PE}}^\delta$, ζ, ϕ, B	15599	16720	56526	57647
σ, λ_{amb}, λ_{ref}, β_0^δ, $\beta_{\bar{R}}^\delta$, $\beta_{w\text{PE}^2}^\delta$, ζ, ϕ, B	15613	16734	16342	17463
σ, λ_{amb}, λ_{ref}, β_0^δ, $\beta_{\bar{R}}^\delta$, β_O^δ, ζ, ϕ, B	<u>**15536**</u>	16657	<u>**15676**</u>	<u>**16797**</u>
σ, λ_{amb}, λ_{ref}, β_0^δ, $\beta_{w\text{PE}}^\delta$, β_n, ζ, ϕ, B	15627	16748	16089	17210
σ, λ_{amb}, λ_{ref}, β_0^δ, $\beta_{w\text{PE}^2}^\delta$, β_n, ζ, ϕ, B	15569	16689	16477	17598
σ, λ_{amb}, λ_{ref}, β_0^δ, β_O^δ, β_n, ζ, ϕ, B	15586	16706	16037	17158

The below table (Table 4) details the fixed values for parameters - i.e. those used when the parameter was not fitted.

S1 Table 4. Parameter values used when the parameter was not fitted)

**Parameter**	**Fixed value**
$\alpha_{\bar{R}}$	0.018 (see [1])
β_0^δ	0
β_n	0
β_O^δ	0
$\beta_{\bar{R}}^\delta$	0
$\beta_{w\mathrm{PE}}^\delta$	0
$\beta_{w\mathrm{PE}^2}^\delta$	0
$\gamma_{w\mathrm{PE}}$	0.61 (see [2])

3 Comparison with alternate models

To further assess the reliability of our novel model, we first compared the results from the POMDP model to those of a set of GLMMs of reaction time. These GLMMS included a random effect of subject, and a fixed effect of stimulus, condition, and within-test experience (trial or average earning rate; and prediction error or previous outcome or squared prediction error). These GLMMs firstly confirmed that the average earning rate better explained within-subject variation in reaction time than trial (ΔBIC=3.958, compared to next best model), and that previous outcome better explained within-subject variation than the prediction error or squared prediction error (ΔBIC=8.372, compared to next best model). Furthermore, in agreement with permutation tests of the parameter estimates, likelihood ratio tests revealed that a more positive previous outcome (LRT=15.262, $p<0.001$) and a higher average earning rate (LRT=22.198, $p<0.001$) significantly predicted greater risk-aversion/'pessimism'.

Then, we assessed how the choices predicted by our model compared to that of an alternative model of choice on the judgement bias task, namely the Bayesian decision model described by Whiteley and Sahani (2008) [3–5]. We adapted this model so that the bias term depended on a baseline bias as well as the average earning rate and previous outcome, and so that the lapse rate differed for ambiguous and reference stimuli, as in our final model, and used the same optimisation procedure for model-fitting. The POMDP model provided a better fit of choice data than the Bayesian decision model (Fig 3; POMDP log-likelihood: 3130 vs. WS log-likelihood: 2919). Jointly, these comparisons support the reliability and utility of our novel model.

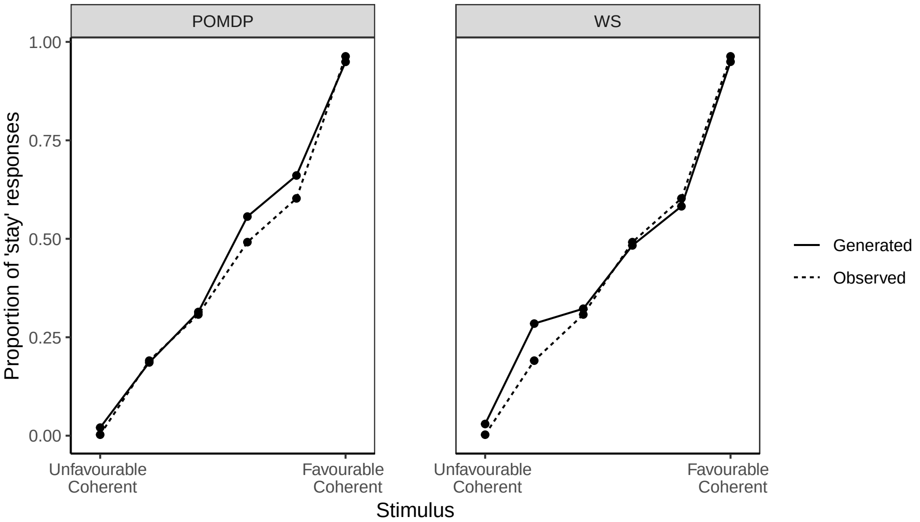

S1 Fig 3. The generated and observed proportion of 'stay' responses in our novel POMDP model (left panel) and an alternative model of judgement bias choice first described by Whiteley and Sahani (right panel).

References

1. Beierholm U, Guitart-Masip M, Economides M, Chowdhury R, Düzel E, Dolan R, et al. Dopamine modulates reward-related vigor. Neuropsychopharmacology. 2013;38(8):1495–1503.
2. Rutledge RB, Skandali N, Dayan P, Dolan RJ. A computational and neural model of momentary subjective well-being. Proceedings of the National Academy of Sciences. 2014;111(33):12252–12257.
3. Whiteley L, Sahani M. Implicit knowledge of visual uncertainty guides decisions with asymmetric outcomes. Journal of Vision. 2008;8(3):1–15.
4. Neville V, King J, Gilchrist ID, Dayan P, Paul ES, Mendl M. Reward and punisher experience alter rodent decision-making in a judgement bias task. Scientific Reports. 2020;10(1):1–14.

5. Iigaya K, Jolivald A, Jitkrittum W, Gilchrist ID, Dayan P, Paul E, et al. Cognitive bias in ambiguity judgements: using computational models to dissect the effects of mild mood manipulation in humans. PloS One. 2016;11(11):e0165840.
